# PPanG: a precision pangenome browser enabling nucleotide-level analysis of genomic variations in individual genomes and their graph-based pangenome

**DOI:** 10.1186/s12864-024-10302-5

**Published:** 2024-04-24

**Authors:** Mingwei Liu, Fan Zhang, Huimin Lu, Hongzhang Xue, Xiaorui Dong, Zhikang Li, Jianlong Xu, Wensheng Wang, Chaochun Wei

**Affiliations:** 1 Department of Bioinformatics and Biostatistics, School of Life Sciences and Biotechnology, Shanghai Jiao Tong University, 800 Dongchuan Road, Shanghai, 200240 China; 2 State Key Laboratory of Crop Gene Resources and Breeding, Institute of Crop Sciences, Chinese Academy of Agricultural Sciences (CAAS), Beijing, 100081 China; 3 College of Agronomy, Anhui Agricultural University, Hefei, 230036 China; 4 National Nanfan Research Institute (Sanya), Chinese Academy of Agricultural Sciences, Sanya, 572024 China

**Keywords:** Genome browser, Graph pangenome, Genome annotation, Comparative pangenomics, Rice

## Abstract

Graph-based pangenome is gaining more popularity than linear pangenome because it stores more comprehensive information of variations. However, traditional linear genome browser has its own advantages, especially the tremendous resources accumulated historically. With the fast-growing number of individual genomes and their annotations available, the demand for a genome browser to visualize genome annotation for many individuals together with a graph-based pangenome is getting higher and higher. Here we report a new pangenome browser PPanG, a precise pangenome browser enabling nucleotide-level comparison of individual genome annotations together with a graph-based pangenome. Nine rice genomes with annotations were provided by default as potential references, and any individual genome can be selected as the reference. Our pangenome browser provides unprecedented insights on genome variations at different levels from base to gene, and reveals how the structures of a gene could differ for individuals. PPanG can be applied to any species with multiple individual genomes available and it is available at https://cgm.sjtu.edu.cn/PPanG.

**Supplementary Information:**

The online version contains supplementary material available at 10.1186/s12864-024-10302-5.

## Background

In traditional genomic studies, a reference genome is selected to represent the genetic materials of a species. However, with the development of DNA sequencing technology, re-sequencing different individual genomes from the same species has gradually revealed much richer genomic diversity. It is now widely accepted that a single reference genome cannot cover the genomic diversity of a species, and using such a single genome as a reference can lead to reference bias [1, 2]. In 2005, Tettelin et al. introduced the concept of pangenome, which refers to the collection of all individual genomes in a species or a population. The pangenome of a species grows much larger than an individual genome as the number of individual genomes increases [3]. For the bacterium, each additional genome incorporated at least 30 new genes to the pangenome. For eukaryotes, for example, the rice pangenome derived from 111 rice accessions has 879Mbps of non-redundant novel sequences, corresponding to about 19,000 novel genes, compared to the reference genome IRGSP-1.0 (*Nipponbare*) [4]. In addition, genes present only in a few individuals of a species can be important, such as those with important functions in plant and animal breeding or human genetic diseases [5–7]. As a result, using a pangenome is of great importance for genomic studies to reduce the bias introduced by using a single individual genome as the reference [8].

The study of pangenome can help to better identify variations among different individuals, including gene presence and absence variation (PAVs), a subtype of structural variations (SVs, defined as sequence variants over 50bps, including deletions, duplications, insertions and translocations). A pangenome can be represented in a linear model or a graph model. Traditionally, linear pangenome combining the reference genome and novel sequences was introduced to deal with pangenome construction and gene PAVs identification [4, 9]. It can inherit and reuse many tools for traditional genomics analysis using an individual genome as the reference. However, the linear pangenome failed to represent some SVs between individuals, such as translocations and inversions [10]. Besides, the novel sequence annotation for linear pangenome construction does not take upstream and downstream genomic context into account, which may result in incomplete gene structures [11]. In a recently published paper, Wang et al. attempted to recover genomic positions of structural variations by embedding novel sequences into a linear pangenome [12]. On the other hand, pangenome graph organizes genomic sequences into a graph in the form of nodes and edges, preserving the sequences and variation information naturally [10]. Different data structures for pangenome graph have been explored and used in a variety of important pangenome construction [13–16]. In 2019, Garrison et al. proposed a graph model called variation graph (VG), a bidirected sequence graph compactly representing variation information across a population, to solve the problem of variation calling in a pangenome [17]. Based on this, Beyer et al. implemented SequenceTubeMap, a pangenome graph browser, to visualize the structure of a variation graph, making it possible to observe nucleotide-level structure variations in a pangenome [18].

The pangenome concept can represent genomic variations in a population. However, the annotation of each individual genome in a pangenome, though in a high demand, is still unavailable for most species, due to the difficulty to produce, visualize and compare them. Typically, annotations of the reference genome are used in genomics studies. However, if we are interested in non-reference regions for a certain individual genome, such as the distributed genes absent in the reference genome, it is difficult to obtain the exact sequence and its annotation. Even if we annotate genomes of all individuals, it is hard to perform the visualization and comparison of genes from different individuals. If a mutation occurs in a genome sequence compared to the reference genome, it is poorly understood whether this gene is absent (which is recognized as a PAV), or how the structure of a gene would differ from its structure in the reference genome if it is still present. Precise gene comparison at nucleotide level can help us to determine how mutations in genomic sequences lead to gene structure variations (gSVs) and PAVs, i.e. the correlation between variations of sequences (SNPs, indels and SVs) and variations of genes (gSVs and PAVs), which is very important to our understanding of variations in pangenomes.

In this study, we constructed a new genome browser that combines graph pangenome visualization and traditional linear genome visualization. Using the rice pangenome previously released by our group [4] as an example, and with the genome annotations for many individual genomes, we showed the design and functions of this novel genome browser. PPanG is designed to visualize not only genomic sequences, but all gene features according to genome annotations, so that variations in both sequences and genes for each individual genome can be observed simultaneously and clearly with their pangenome.

## Results

### Annotation of individual genomes in our rice pangenome

We annotate 114 individual genomes (including reference genome IRGSP-1.0). Statistics of the genome annotations are summarized in Fig. S1. The average size of rice genomes is 409.6 Mbps, while several cultivated accessions (such as TG12 and TG54) and all wild rice accessions are significantly larger than the other individuals. The average number of genes annotated is 36,474 per genome, and the numbers of genes in larger genomes also tend to be larger. The average time to annotate a single individual genome is 275 core hours. By running in parallel, the annotation of all individual rice genomes can be done in one to two weeks.

As there are several outliers, further evaluation is conducted by aligning these genomes to the nt database. We find that Suijing18 contains many non-Oryza matches (Table S1), such as *Pseudomonas*, *Kosakonia* and *Herbaspirillum*. This indicates that Suijing18 suffers from contamination. Therefore, in the following steps, we no longer take Suijing18 into consideration, and the total number of genomes in PPanG is 113.

### Rice pangenome graph construction

All rice genomes are collected and partitioned into 12 chromosomes. Then pangenome graphs are constructed separately for each chromosome using MC and PGGB. PGGB provides diagnostic visualization of pangenome graph by default. The full version of visualization results is shown in Fig. S2, which present high synteny in all samples. Subsequently, these graphs are converted to VG format for visualization in PPanG.

MC and PGGB adopt different strategies for pangenome graph construction. In some regions (Fig. S3), especially for those with simple repeats, PGGB tends to wrap the genome paths with consecutive loops, while MC splits the paths into separate nodes. Although the visualization of MC is less complex and easier to read, there is no sufficient evidence to determine which one is a better representation of the pangenome graph. Therefore, we decide to keep both graphs for users to choose and set MC graph as the default in PPanG.

### Overview of PPanG

**Fig. 1:**
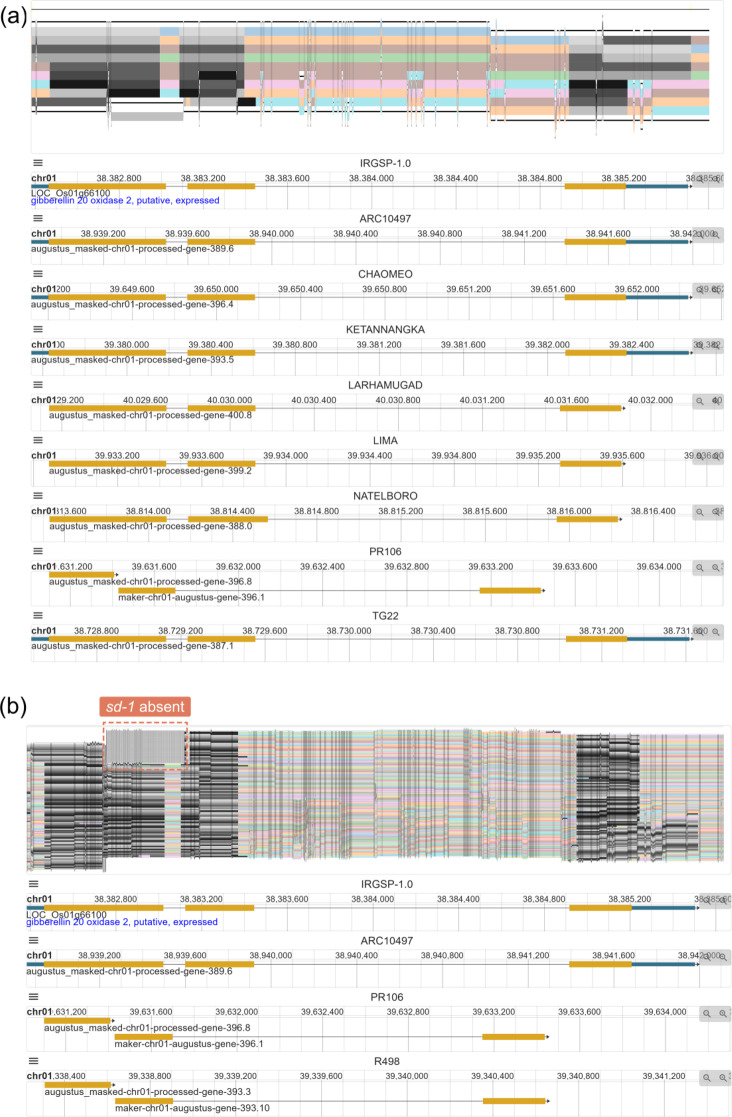
Example views of PPanG. The region with gene *sd-1* is shown by default. The pangenome graph is compressed and texts are hidden. The colored paths represent genome tracks and the gray bars over them represent gene features. The traditional linear genome views (JBrowse2) are embedded below. **(a)** The pangenome graph with only nine representative accessions, and their corresponding individual genome annotations. **(b)** The pangenome graph containing all 113 individual genomes. All accessions can be divided into two groups based on the presence/absence status of *sd-1* gene. The *sd-1* absent group is marked in the figure, while the other genomes belong to *sd-1* present group. Two examples from each group are visualized in JBrowse2 below

Our pangenome browser PPanG is deployed in https://cgm.sjtu.edu.cn/PPanG combining a graph-based pangenome and many individual genomes. The *sd-1* gene region is selected and visualized by default (Fig. 1). In graph view, genome sequences are drawn as paths in colors by SequenceTubeMap. We developed a method (see Methods for more details) to visualize all gene features over the genome tracks to compare these genes at nucleotide level. As the raw pangenome graph could be exceedingly long if each base was visualized, it can be compressed by clicking the “Compress” button. By default, only nine representative genomes are visible (Fig. 1a) for simplicity, and users are free to add any individual of interest into the pangenome graph. It is possible to visualize all genome tracks (Fig. 1b), but is not recommended as a pangenome graph can be very large and complicated for visualization. Traditional linear genome browser in JBrowse2 [19] is provided below the SequenceTubeMap view. The reference genome IRGSP-1.0 is shown by default, and other genomes can be added by double clicking the paths in the pangenome graph. We ensure each gray bar representing an exon in SequenceTubeMap view is approximately aligned to the gene feature in JBrowse2, which may serve as a reference for interpretation.

To make it more user-friendly and easier to get started, we have created views for 69 genes favorable for breeding (Table S2) from China Rice Data Center (https://www.ricedata.cn/). We will take *sd-1* gene as an example to show how to interpret the PPanG visualization.

### Interpretation of PPanG visualization in *sd-1* gene region

**Fig. 2:**
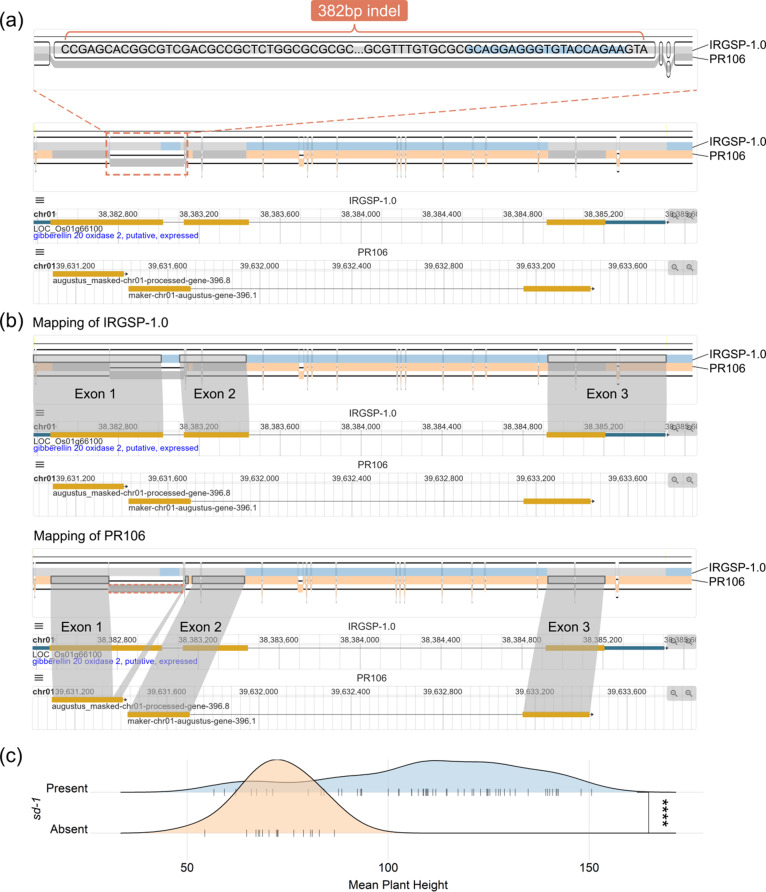
Visualization of *sd-1* gene region in the reference genome IRGSP-1.0 and another genome PR106. The path of PR106 skips a long 382 bp node. The SequenceTubeMap view clearly shows that this skipped node in PR106 represents a deletion in exons 1, 2 and intron 1 of gene OS01g66100 in IRGSP-1.0 and two genes are annotated in PR106. **(a)** Original visualization in PPanG. The deletion of 382 bp node is locally amplified. **(b)** Detailed mapping of IRGSP-1.0 and PR106 in graph view and linear view. **(c)** Ridgeline plot of plant height data between *sd-1* present and absent groups. The p-value = 1.7e-6. Wilcoxon rank-sum test is performed to test the significance of difference

The region for “green revolution gene” *sd-1* (LOC_Os01g66100) is set for visualization by default because it is one of the most well-known distributed genes. Previous studies have shown that in some accessions there is a deletion of about 383 bps in exons 1, 2 and intron 1, indicating the absence of *sd-1* and leading to dwarfism [20, 21]. This mutation can be clearly observed by PPanG. We take the reference genome IRGSP-1.0 and *sd-1* absent accession PR106 and visualize the *sd-1* region (Fig. 2a). As shown in the figure, the bottom path PR106 skips a 382 bp node compared to IRGSP-1.0, inducing the deletion of exons 1, 2 and intron 1. The visualization by PPanG shows that *sd-1* gene is absent in PR106, as this region in PR106 is annotated as two different genes (Fig. 2b). The deletion in genome sequence greatly changes the gene structure and causes a PAV in PR106. In Fig. 1b, all accessions can be divided into two groups based on whether this 382 bp node is skipped (considered as *sd-1* absence). To validate this conclusion, their plant height data are collected in Fig. 2c. The Wilcoxon rank-sum test shows that the *sd-1* present group is significantly higher than the absent group (p-value < 1e-5), which is consistent with previous researches. This example demonstrates the advantage of visualization of variations at both sequence and gene levels in PPanG.

### Locating of novel sequences

Chromosomal location information is necessary to visualize any sequence, especially for novel sequences which are absent in the reference genome. To make the process of locating novel sequences easier, a BLAT service is embedded in PPanG. BLAT database is composed of all 113 rice genomes and loaded in memory for a faster response speed. After inputting a query sequence, the BLAT server will automatically find matches and provide aligned regions in PPanG. Each genome can be considered as reference in PPanG, so the visualization of novel sequences can be done easily.

### Nucleotide-accurate gene comparison for our rice pangenome

PPanG makes it possible to observe PAVs and gSVs directly by comparing the gene structures from different individuals. We know that variations are universal in organisms’ genomes. However, these variations of sequences do not necessarily lead to the variations of genes. In pangenomes, core genes, which are present in all individuals, should be more conserved than distributed/dispensable genes, which are present only in some individuals. Next, we will take a close look at the variations within different types of genes.

### Comparison of genes present in all individuals (core genes)

**Fig. 3:**
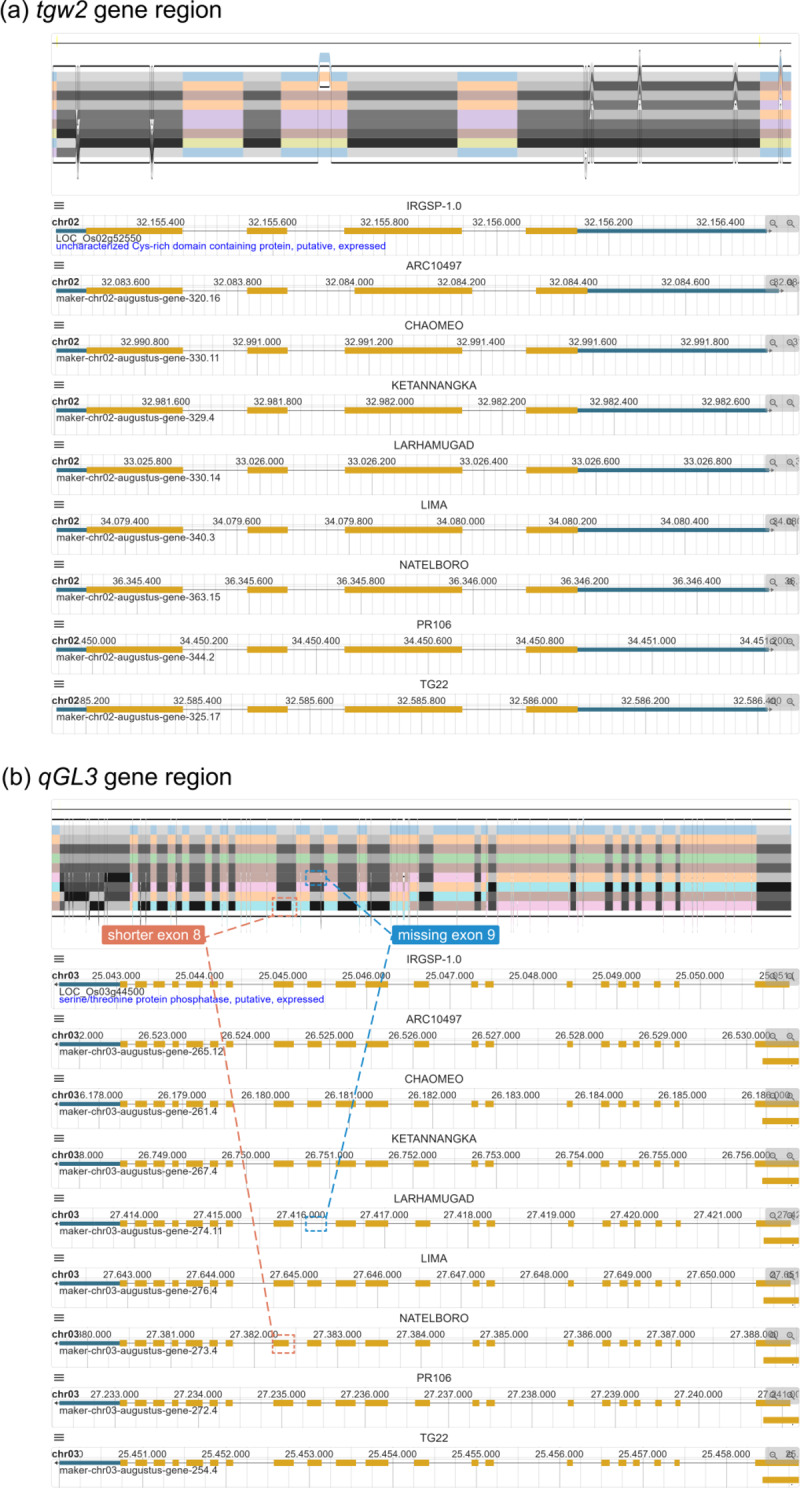
Visualization of core gene regions in compressed view for nine representative genomes. **(a)** PPanG visualization of *tgw2* gene region. **(b)** PPanG visualization of *qGL3* gene region. The PAVs of gene elements are marked in NATELBORO and LARHAMUGAD. Although mutations occur in genome sequences, the splice sites from different individuals are identical. It illustrates that variations in genome sequence do not necessarily change the gene structure greatly

Taking the grain weight gene *tgw2* (LOC_Os02g52550) as an example, overall structure of this gene region is visualized in Fig. 3a in compressed view. There are nine genomes and four gene features present in this region. In traditional linear genome browsers, these gene structures are nearly the same, but it is ambiguous due to slight shifts caused by indels. Instead, as the SequenceTubeMap view shows, both two ends of each exon in gray are perfectly aligned to each other, illustrating that all splice sites are identical in different individuals. These gene structures are the same as *tgw2* in IRGSP-1.0, i.e., *tgw2* is a core gene present in these nine genomes. It is important to point out that these nine sequences are not identical, as several mutations occur in both exons and introns. However, these mutations do not lead to great variations in the gene structure, presumably because they are substitutions or indels whose lengths are multiples of three, which means the open reading frame (ORF) does not shift. Another example is the visualization of *qGL3* (LOC_Os03g44500) gene region in Fig. 3b. The splice sites of gene elements are almost the same except the shorter exon 8 in NATELBORO and missing exon 9 in LARHAMUGAD, which indicates the PAV of gene elements. PPanG visualization in core gene regions reveals that the genome sequences of highly conserved gene regions are not necessarily identical, but these base mutations may not affect the gene structure significantly. This situation is similar for genes like *HMS1* (LOC_Os03g12030), *PROG1* (LOC_Os07g05900), *DEP2* (LOC_Os07g42410), *xa13* (LOC_Os08g42350) (Fig.S3), etc.

### Comparison of genes not present in all individuals (distributed genes)

**Fig. 4:**
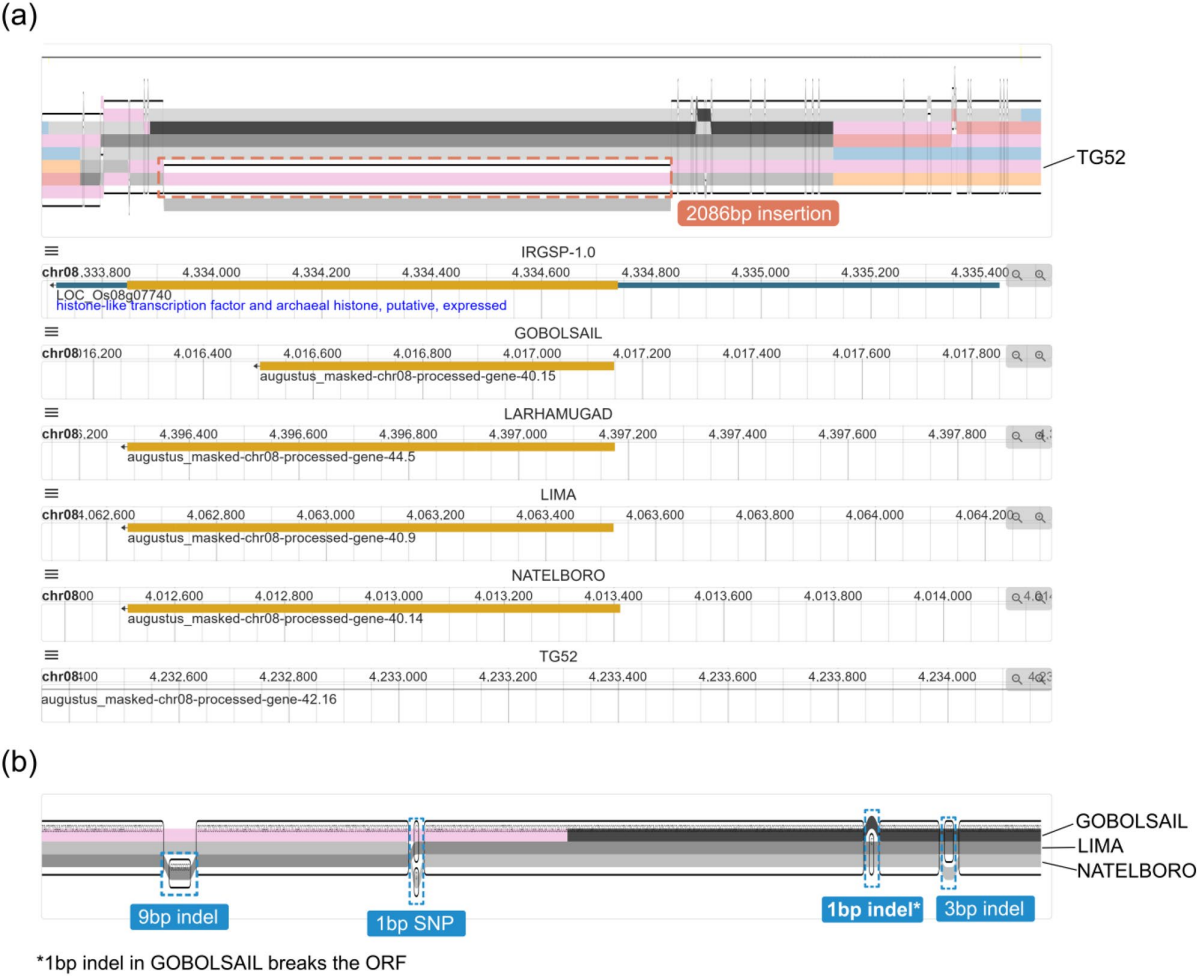
Visualization of distributed gene *Hd5* in PPanG. **(a)** The overview of gene structure in six genomes in compressed view. There is a 2,086 bp indel in TG52 genome, leading to the absence of *Hd5*. Besides, the exon in GOBOLSAIL is shorter than other genomes. **(b)** The detailed visualization of variations in GOBOLSAIL, LIMA and NATELBORO. There are four variations in this region, but only the 1 bp indel in GOBOLSAIL breaks the ORF and causes the shortness of the exon

In other cases, there may be great differences in gene structures, especially in distributed genes. The gene *sd-1* mentioned above is a typical distributed gene. Another case for distributed genes is heading date gene *Hd5* (LOC_Os08g07740). Six rice genomes are selected for visualization in Fig. 4. As the linear genome browsers show, exons in LARHAMUGAD, LIMA and NATELBORO are identical to the reference genome, while the exon is much shorter in GOBOLSAIL and absent in TG52. Figure 4a shows that a 2,086 bp insertion associates with a PAV in TG52. For GOBOLSAIL, the detailed variation is shown in Fig. 4b. The 1 bp indel which breaks the ORF explains the shortness of the exon in GOBOLSAIL. Figure 4 illustrates the relationship between indels and gSVs/PAVs clearly.

**Fig. 5:**
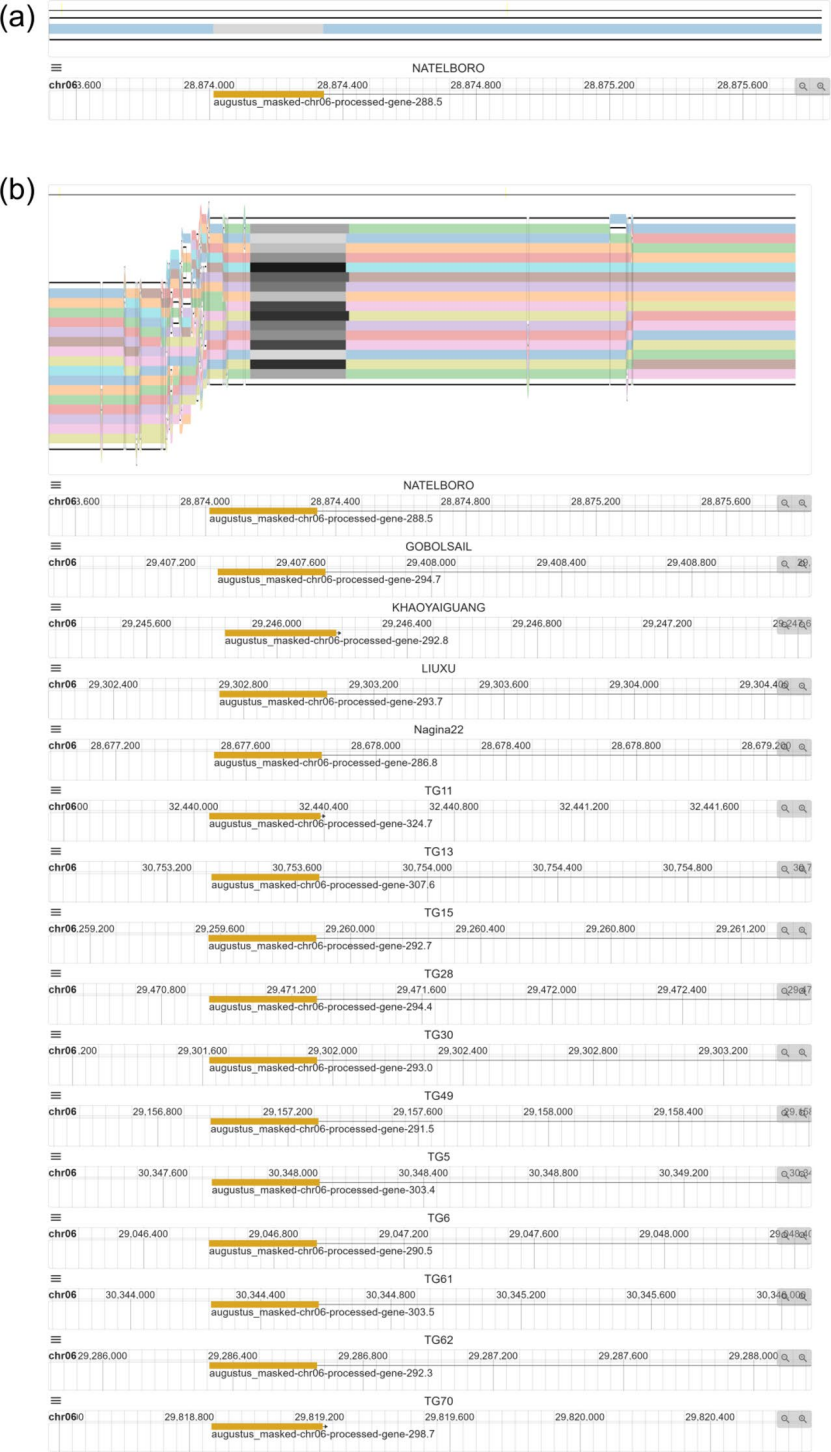
Visualization of distributed genes *Xa7* in compressed view. **(a)** Nine representative genomes are selected in the pangenome graph but only NATELBORO is shown in this gene region. It indicates that *Xa7* is present in NATELBORO and absent in the other eight genomes. **(b)** All genomes are selected in the SequenceTubeMap view. There are 16 tracks aligned in this region in total. It suggests that *Xa7* is present in these 16 genomes and absent in the other 97 genomes

We also consider a special case about 1,345 bp bacterial blight resistance gene *Xa7*, which is reported to be absent in the reference genome [22, 23]. To locate this gene region, we run the BLAT embedded in PPanG to search for *Xa7* sequence in nine representative genomes and find only one match in the region “NATELBORO.chr06:28873554–28874897” with 1,344 matches and one gap. The BLAT result indicates that *Xa7* is present in NATELBORO. Then this region is provided for PPanG for visualization (Fig. 5a). There is only one node and one path in the pangenome graph, which means that there are no similar sequences aligned in the other eight genomes. So *Xa7* is unique to NATELBORO. There are 16 paths out of the total 113 rice genomes are aligned in this region, and the full visualization is shown in Fig. 5b. The visualization in PPanG suggests that *Xa7* is present in these 16 genomes and absent in the other 97 genomes. This special case clearly demonstrates how to locate and visualize a distributed or unique gene which is absent even in the reference genome.

In summary, these examples illustrate different gSVs between individuals in core and distributed genes, which provide strong evidence to support our understandings of gene variations in pangenomes.

### Comparison of annotations in a targeted region

PPanG can serve as a tool to compare different annotation datasets. For example, we show detailed comparison between RGAP7 (Michigan State University Rice Genome Annotation Project Release 7 [24, 25], MSU RGAP7) and our annotations in Fig. S4. Among all the built-in genes, *GS3* on chromosome 3, *OsPTS1* on chromosome 8 and *Xa7* (Fig. 4) are absent in RGAP7 but present in our dataset. Conversely, there are two genes absent in our annotations, but present in RGAP7: *OsRCI2-5* (LOC_Os03g17790) on chromosome 3 and *pms3* (LOC_Os12g36030) on chromosome 12. In general, the gene structures of our annotations are consistent with RGAP7, although there may be occasional differences in the numbers and lengths of gene features.

### Comparison with existing pangenome browsers

Besides PPanG, there are several existing pangenome browsers currently, but their focuses are different. The brassica browser proposed by Golicz et al. in 2016 [11], includes annotations on a linear pangenome, which is limited to a rough linear visualization of nodes at gene level. Panache [13] focused on variations in blocks especially at gene level based on a linear pangenome. Another pangenome browser Momi-G [26] also adopted SequenceTubeMap for visualization. It focused on the visualization of genomic variations, especially structure variations, though annotations for each individual genome were not shown in the same page with the graph-based pangenome. PPanG is the first browser to enable the nucleotide-level visualization of multiple individual genomes together with their graph-based pangenome. It can accelerate our understanding about genomic variations both at a population level and individual genome level.

## Discussion and conclusion

In this paper, we take rice pangenome as an example, and utilize our pangenome browser PPanG to visualize a graph-based pangenome and individual genomes with their annotations. Based on PPanG, we firstly show the nucleotide-level gene comparison in core genes and distributed genes, and reveal the potential correlation between sequence variations and gene variations. Although PPanG is designed to visualize the individual genomes and a pangenome of them for rice, it can be applied to other species given the genome sequence data and their annotations. PPanG presents genomic variations using pangenomes and individual genome annotations from a new perspective, making it a powerful tool to advance our understandings of genomic variations in a species or a population.

However, the development of pangenome graph visualization is still in progress with limitations and requires further improvement. First, the algorithm for graph construction is not mature yet, as nodes may become intricately connected, making the graph visualization chaotic and peculiar. Moreover, whether simpler pangenome graph visualization is better or not is under discussion. Some people believe that pangenome graph visualization is supposed to contain all variations, while others consider that too many variations will drown out the signals of important variations. Therefore, it may be a better choice for genome browser users to decide the resolution of pangenome graph visualization. Proper approaches to merge different nodes and reorganize the original graph structure are needed. Besides, there is currently a lack of an efficient method to split the graph and extract a subgraph of a specific region, which is required to visualize a custom region in a timely manner. Another challenge is the scalability of pangenome graph construction. The issue of how to quickly and accurately add new individuals to an existing pangenome graph remains a thorny problem to be solved. Finally, the current method to combine the graph views and linear views has its limitation. Especially, the coordinates of the graph pangenome are shared by different genomes and not evenly distributed due to the insertions and deletions. The mapping of coordinates will be better if the coordinate systems of linear views are flexible and exactly aligned to the graph view. Although the pangenome graph visualization has some limitations at present, this paper demonstrates the vast potential of using graph-based model to depict pangenomes.

## Methods

**Fig. 6:**
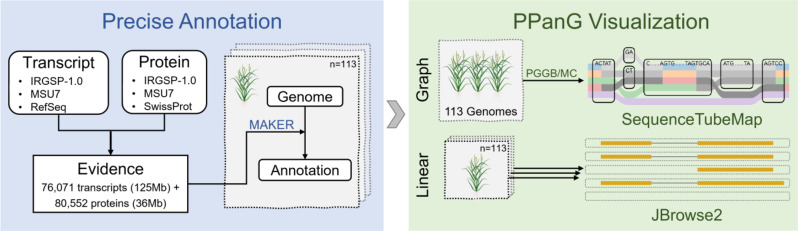
Overview of PPanG pipeline. First, all rice genomes are annotated by MAKER with transcript and protein evidences. These annotations and the pangenome data are provided to PPanG. The pangenome graph is visualized by SequenceTubeMap, while individual genomes are visualized by JBrowse2

Pangenome data and genome annotations of all individuals are needed in PPanG. Figure 6 summarizes the pipeline of PPanG.

### Rice pangenome data

Genome data used in this study are from the rice pangenome previously constructed by our group, composed of 105 *Oryza sativa* (OS) accessions and 6 *Oryza rufipogon* (OR) accessions (113 samples in total, with three samples from the same accession IR64), with the raw data reaching 3 TBs [4]. The rice reference genome IRGSP-1.0 is also included. Nine accessions from representative rice populations are selected, and corrected reads and polished contigs are used to fill the gaps. These nine high-quality genomes, including gapless genomes NATELBORO, PR106, LARHAMUGAD, LIMA, KETANNANGKA and IRGSP-1.0, are considered as default tracks in PPanG.

### Genome annotation

These rice genomes are annotated by genome annotation pipeline MAKER/2.31.9 with OpenMPI. Transcript evidences (76,071 transcripts, 125 MBs) used in MAKER are collected from *Oryza* mRNAs in NCBI nucleotide database (https://www.ncbi.nlm.nih.gov/nuccore), and protein homology evidences (80,552 proteins, 36 MBs) are collected from *Oryza* proteins in NCBI protein database (https://www.ncbi.nlm.nih.gov/protein). These evidences are clustered by cd-hit [27, 28] at 90% identity level to build a non-redundant evidence set. The model species for repeat masking and Augustus gene prediction is set to rice. The threshold for unsupported gene prediction is set to 1. The other configurations for MAKER remain default.

### Pangenome graph construction and visualization

Pangenome graphs are built by two graph builders: the Pangenome Graph Builder/v0.5.3-19-g507fc04 (PGGB) [29] and Minigraph-Cactus/v2.2.2 (MC) [30]. Both two graph builders are designed to integrate multiple steps, including genome alignment, graph induction and normalization into a pipeline. After constructing the raw pangenome graph, VG [17] provides various tools to deal with the pangenome graph and convert it into VG format. These pangenome graphs are provided to PPanG in the SequenceTubeMap view.

### Nucleotide-level visualization of multi-annotations

**Fig. 7:**
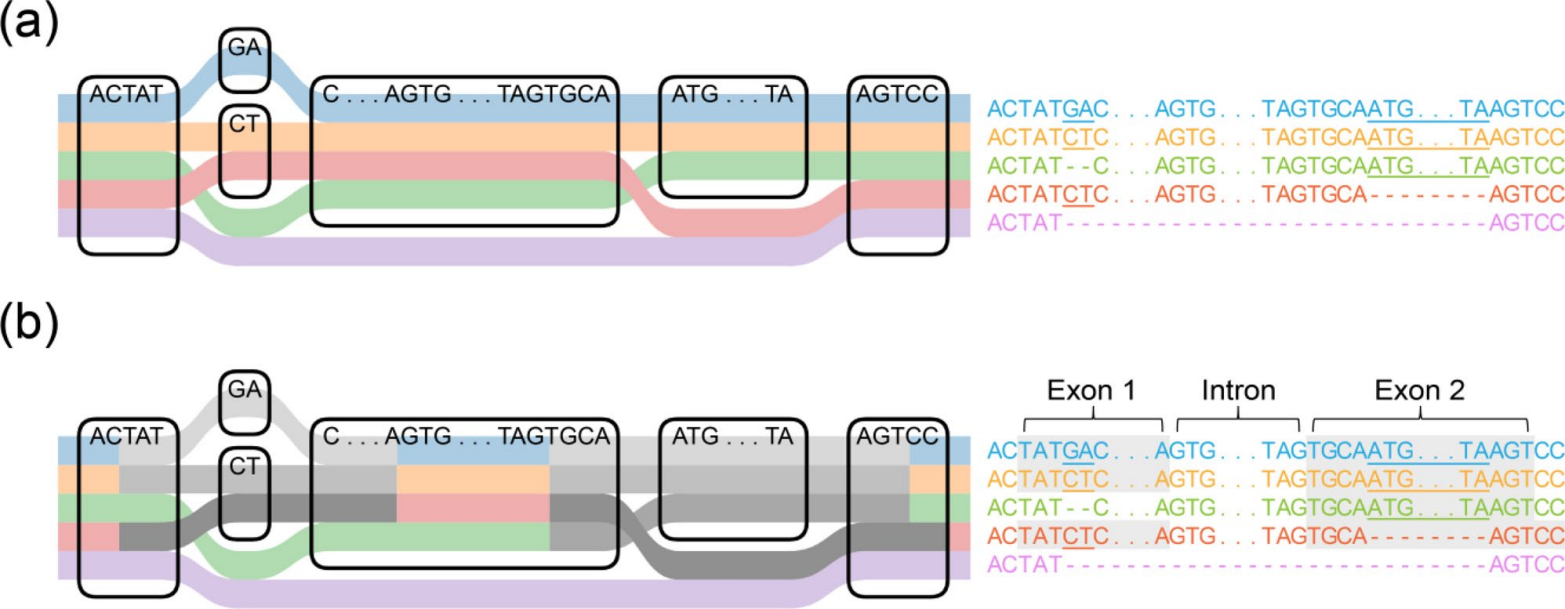
A simple demo of pangenome graph about precise gene comparison in PPanG. (**a**) The pangenome sequence graph only. Source sequences are presented on the right and variations are underlined. (**b**) The pangenome sequence graph with gene features visualized. Two exons are drawn in gray bars over genome tracks according to genome annotations. As the figure shows, the junctions of exon 1 from sequence 1, sequence 2 and sequence 4 are identical, although there is a substitution from “GA” to “CT”. In sequence 3, exon 2 is identical but exon 1 is absent due to the deletion, which represents a gSV. There is not any gray bar in sequence 5, indicating that all gene features are absent due to the large deletion, which represents an absent variation. Notably, the second gray bar of sequence 4 appears longer than the others, but actually exon 2 of sequence 4 is shorter because of the missing node of “ATG…TA”, which may be counterintuitive

PPanG is the first genome browser for precise visualization of multi-annotations at nucleotide level. As the graph model is composed of nodes and edges, it is natural to visualize the pangenome graph by drawing each node and connecting them with edges. Figure 7a shows a simple graph demo with various substitutions and indels in SequenceTubeMap. However, SequenceTubeMap cannot simultaneously visualize multiple genome annotations. To compare different genes, in PPanG, genome annotations are visualized by drawing gene features in gray over the sequence tracks with coordinate conversion and alignment (Fig. 7b). This pattern illustrates the gene structure: The colored region before the initial exon or after the ending exon represents intergenic region, and the region between two exons from a same gene represents an intron. By nucleotide-level gene comparison, it is obvious to identify PAVs (absence of the gray bar represents absence of the feature/gene) and gSVs (number, locations and lengths of the gray bars represent the gene structure) in PPanG.

### Coloration patterns in pangenome graph visualization

In PPanG, genome sequences are colored in light colors by default, and exons will be marked in gray if users choose to show exons (Fig. 7). Whereas, some users feel more comfortable to tone other sequences down by coloring them in gray except for the exons, which means converting the coloration pattern. Therefore, PPanG provides alternative coloration options to meet different users’ preferences.

### PPanG implementation

PPanG is composed of graph pangenome browser view implemented by SequenceTubeMap (https://github.com/vgteam/sequenceTubeMap) and paralleling linear genome browser views implemented by JBrowse2 embedded components (https://www.jbrowse.org/jb2/docs/embedded_components/). The codes are based on Javascript and React (https://react.dev). For graph browser, as SequenceTubeMap couldn’t handle visualization of multi-annotations, we modified the codes to add or override many features, including annotation data extraction, annotation coordinates conversion based on nucleotide sequences and region navigating by searching gene IDs. For linear browser, as one JBrowse2 window is limited to one reference genome, we decide to use multiple JBrowse2 components for individual genomes, and align their start coordinates according to the pangenome graph. The pangenome graph stores the whole alignment information with nodes and paths, and provides the corresponding coordinates of aligned sequences in terms of each individual’s coordinates. However, the coordinates of pangenome graph are interrupted by indels while the coordinates for individual genomes are not, so these coordinates cannot be perfectly aligned to each other. Besides, we implement a feature for real-time synchronization, which means the change of SequenceTubeMap region will send a message to JBrowse2 views to navigate them to the target region. These unique features highlight PPanG from other pangenome browsers.

### Electronic supplementary material

Below is the link to the electronic supplementary material.

Supplementary Material 1

Supplementary Material 2

Supplementary Material 3

Supplementary Material 4

Supplementary Material 5

Supplementary Material 6

Supplementary Material 7

## Data Availability

All code can be accessed in https://github.com/SJTU-CGM/PPanG/. All data mentioned in this paper can be accessed in https://cgm.sjtu.edu.cn/PPanG/.
