# Supplementary material for: PPanG: a precision pangenome browser enabling nucleotide-level analysis of genomic variations in individual genomes and their graph-based pangenome
Source: BMC Genomics. 2024 Apr 24;25:405. doi: 10.1186/s12864-024-10302-5 (PMC11044437; doi:10.1186/s12864-024-10302-5)
Supplement: Supplementary file 3 — Supplementary Material 3 [file 12864_2024_10302_MOESM3_ESM.pdf]

Fig.S3 Example views of core gene regions *HMS1* (LOC_Os03g12030), *PROG1* (LOC_Os07g05900), *DEP2* (LOC_Os07g42410) and *xa13* (LOC_Os08g42350) in PPanG. These visualizations reveal that the genome sequences of highly conserved gene regions are not necessarily identical, but these base mutations do not affect the gene structure significantly.

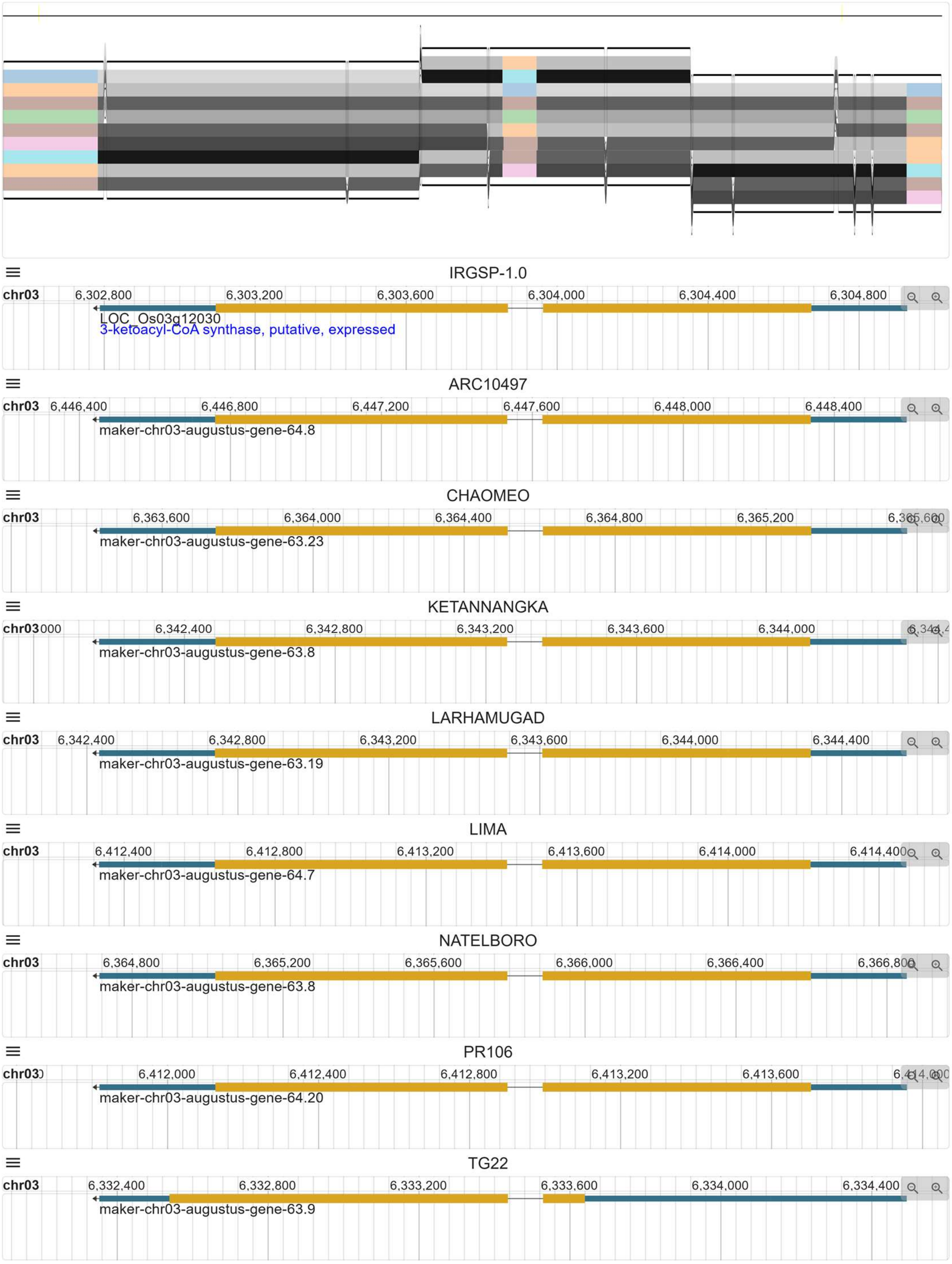

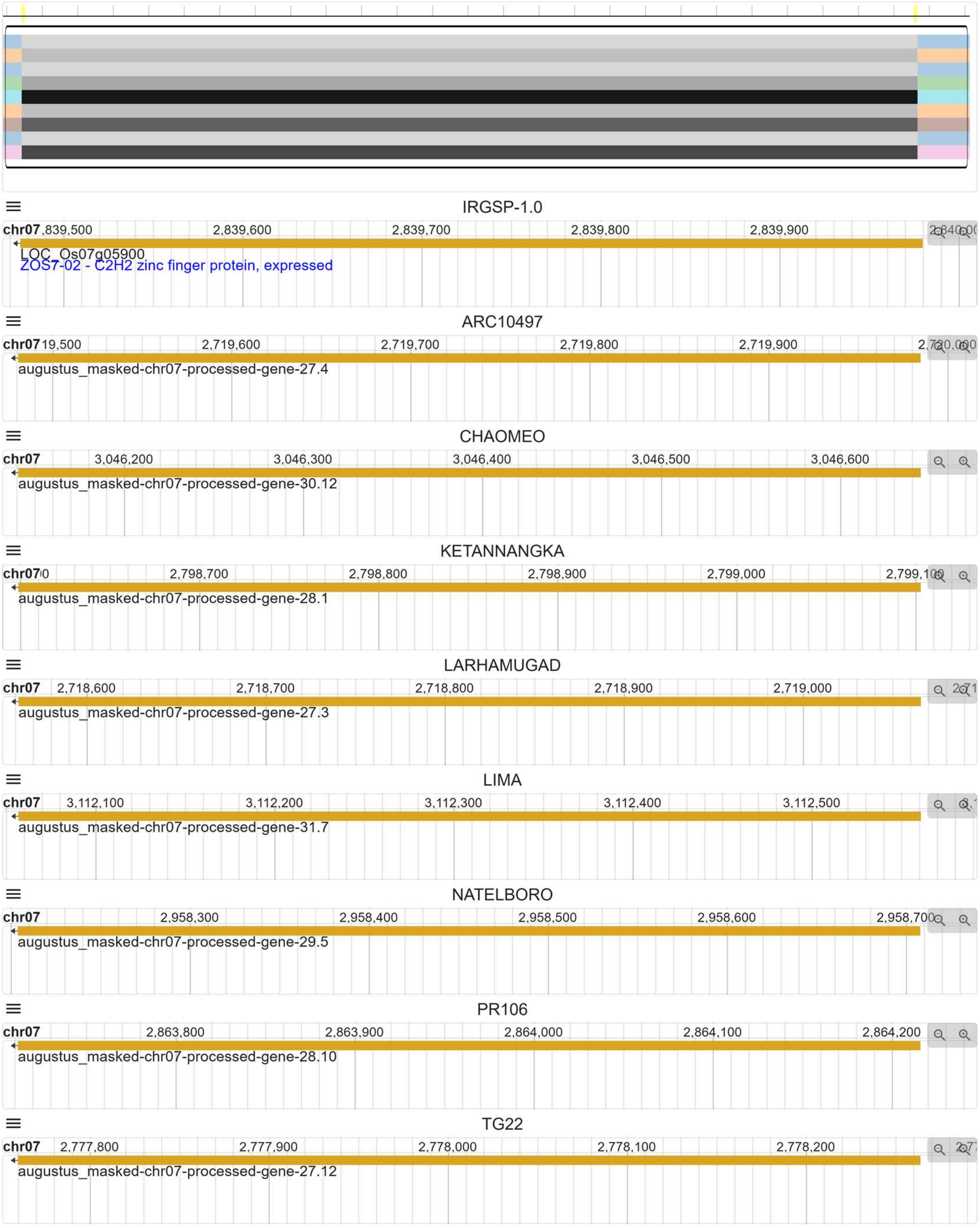
IRGSP-1.0
chr07
2,839,600
2,839,700
2,839,800
2,839,900
LOC_Os07g05900
ZOS7-02 - C2H2 zinc finger protein, expressed
ARC10497
chr07
2,719,600
2,719,700
2,719,800
2,719,900
augustus_masked-chr07-processed-gene-27.4
CHAOMEO
chr07
3,046,200
3,046,300
3,046,400
3,046,500
3,046,600
augustus_masked-chr07-processed-gene-30.12
KETANNANGKA
chr07
2,798,700
2,798,800
2,798,900
2,799,000
augustus_masked-chr07-processed-gene-28.1
LARHAMUGAD
chr07
2,718,600
2,718,700
2,718,800
2,718,900
2,719,000
augustus_masked-chr07-processed-gene-27.3
LIMA
chr07
3,112,100
3,112,200
3,112,300
3,112,400
3,112,500
augustus_masked-chr07-processed-gene-31.7
NATELBORO
chr07
2,958,300
2,958,400
2,958,500
2,958,600
augustus_masked-chr07-processed-gene-29.5
PR106
chr07
2,863,800
2,863,900
2,864,000
2,864,100
2,864,200
augustus_masked-chr07-processed-gene-28.10
TG22
chr07
2,777,800
2,777,900
2,778,000
2,778,100
2,778,200
augustus_masked-chr07-processed-gene-27.12

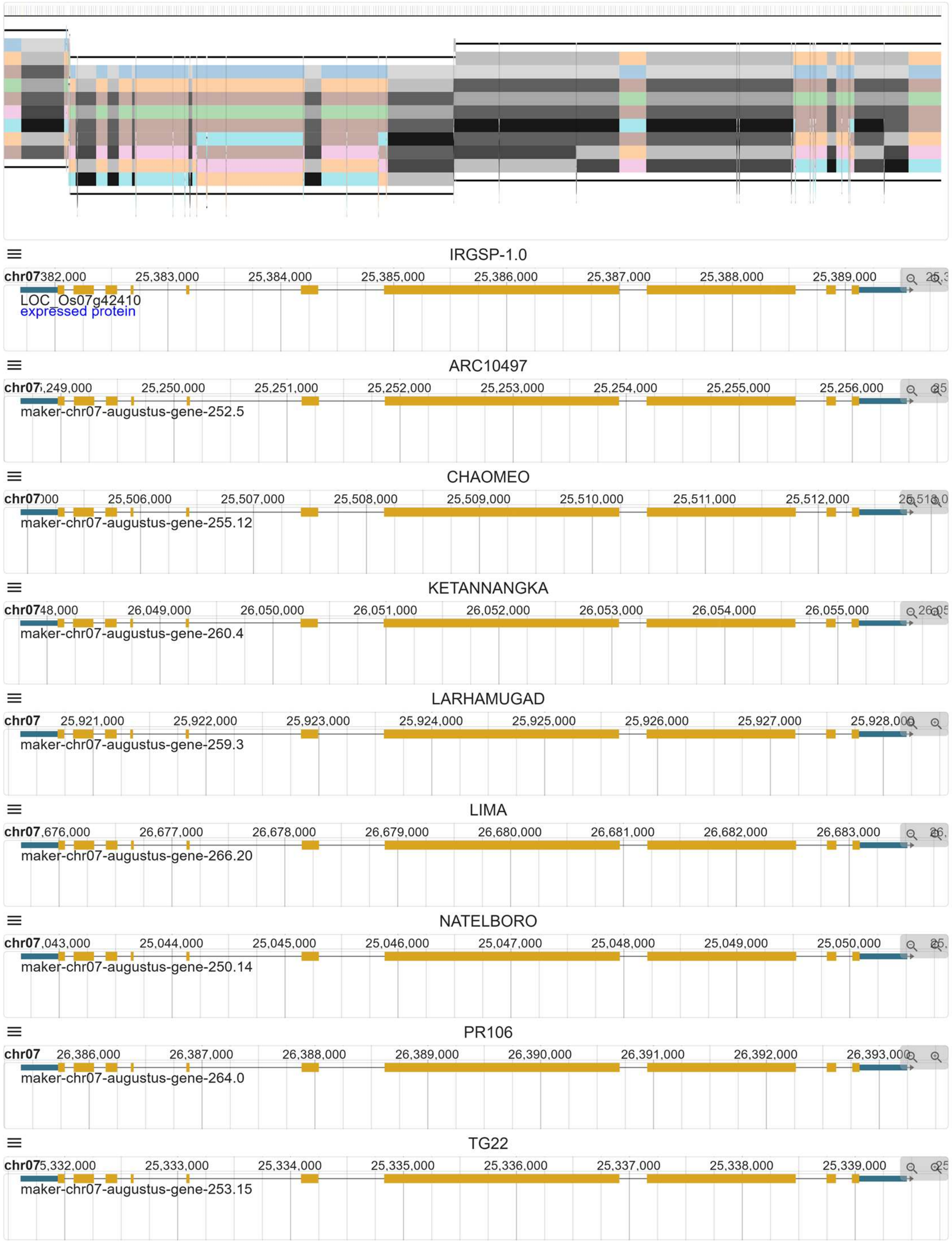

IRGSP-1.0
chr07
382,000
25,383,000
25,384,000
25,385,000
25,386,000
25,387,000
25,388,000
25,389,000
LOC_Os07g42410
expressed protein
ARC10497
chr07
25,250,000
25,251,000
25,252,000
25,253,000
25,254,000
25,255,000
25,256,000
maker-chr07-augustus-gene-252.5
CHAOMEO
chr07
25,506,000
25,507,000
25,508,000
25,509,000
25,510,000
25,511,000
25,512,000
maker-chr07-augustus-gene-255.12
KETANNANGKA
chr07
48,000
26,049,000
26,050,000
26,051,000
26,052,000
26,053,000
26,054,000
26,055,000
maker-chr07-augustus-gene-260.4
LARHAMUGAD
chr07
25,921,000
25,922,000
25,923,000
25,924,000
25,925,000
25,926,000
25,927,000
maker-chr07-augustus-gene-259.3
LIMA
chr07
,676,000
26,677,000
26,678,000
26,679,000
26,680,000
26,681,000
26,682,000
26,683,000
maker-chr07-augustus-gene-266.20
NATELBORO
chr07
,043,000
25,044,000
25,045,000
25,046,000
25,047,000
25,048,000
25,049,000
25,050,000
maker-chr07-augustus-gene-250.14
PR106
chr07
26,386,000
26,387,000
26,388,000
26,389,000
26,390,000
26,391,000
26,392,000
maker-chr07-augustus-gene-264.0
TG22
chr07
5,332,000
25,333,000
25,334,000
25,335,000
25,336,000
25,337,000
25,338,000
25,339,000
maker-chr07-augustus-gene-253.15

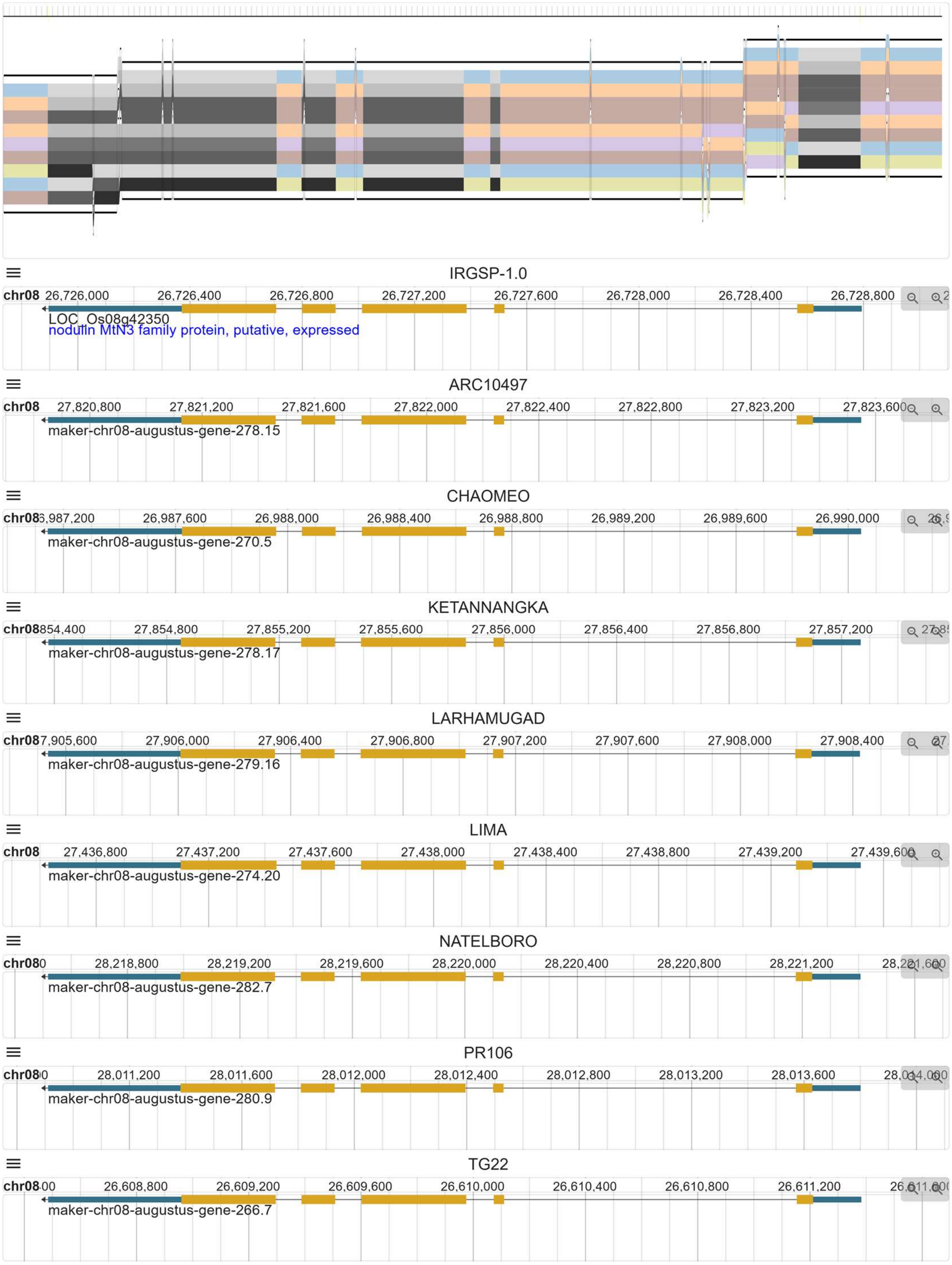
IRGSP-1.0
chr08
26,726,000
26,726,400
26,726,800
26,727,200
26,727,600
26,728,000
26,728,400
26,728,800
LOC_Os08g42350
nodulin MtN3 family protein, putative, expressed
ARC10497
chr08
27,820,800
27,821,200
27,821,600
27,822,000
27,822,400
27,822,800
27,823,200
27,823,600
maker-chr08-augustus-gene-278.15
CHAOMEO
chr08
26,987,600
26,988,000
26,988,400
26,988,800
26,989,200
26,989,600
26,990,000
maker-chr08-augustus-gene-270.5
KETANNANGKA
chr08
27,854,800
27,855,200
27,855,600
27,856,000
27,856,400
27,856,800
27,857,200
maker-chr08-augustus-gene-278.17
LARHAMUGAD
chr08
27,906,000
27,906,400
27,906,800
27,907,200
27,907,600
27,908,000
27,908,400
maker-chr08-augustus-gene-279.16
LIMA
chr08
27,436,800
27,437,200
27,437,600
27,438,000
27,438,400
27,438,800
27,439,200
maker-chr08-augustus-gene-274.20
NATELBORO
chr08
28,218,800
28,219,200
28,219,600
28,220,000
28,220,400
28,220,800
28,221,200
maker-chr08-augustus-gene-282.7
PR106
chr08
28,011,200
28,011,600
28,012,000
28,012,400
28,012,800
28,013,200
28,013,600
maker-chr08-augustus-gene-280.9
TG22
chr08
26,608,800
26,609,200
26,609,600
26,610,000
26,610,400
26,610,800
26,611,200
maker-chr08-augustus-gene-266.7
