# Supplementary material for: PPanG: a precision pangenome browser enabling nucleotide-level analysis of genomic variations in individual genomes and their graph-based pangenome
Source: BMC Genomics. 2024 Apr 24;25:405. doi: 10.1186/s12864-024-10302-5 (PMC11044437; doi:10.1186/s12864-024-10302-5)
Supplement: Supplementary file 4 — Supplementary Material 4 [file 12864_2024_10302_MOESM4_ESM.pdf]

Fig.S4 Comparison of MSU7 and our annotations in PPanG. *GS3* on chromosome 3 and *OsPTS1* on chromosome 8 and *Xa7* (Fig.4) are absent in MSU7 but present in our dataset. *OsRCI2-5* (LOC_Os03g17790) on chromosome 3 and *pms3* (LOC_Os12g36030) on chromosome 12 are present in our dataset but absent in MSU7. In general, the gene structures of our annotations are consistent with MSU7, although there may be occasional differences in the numbers and lengths of gene features.

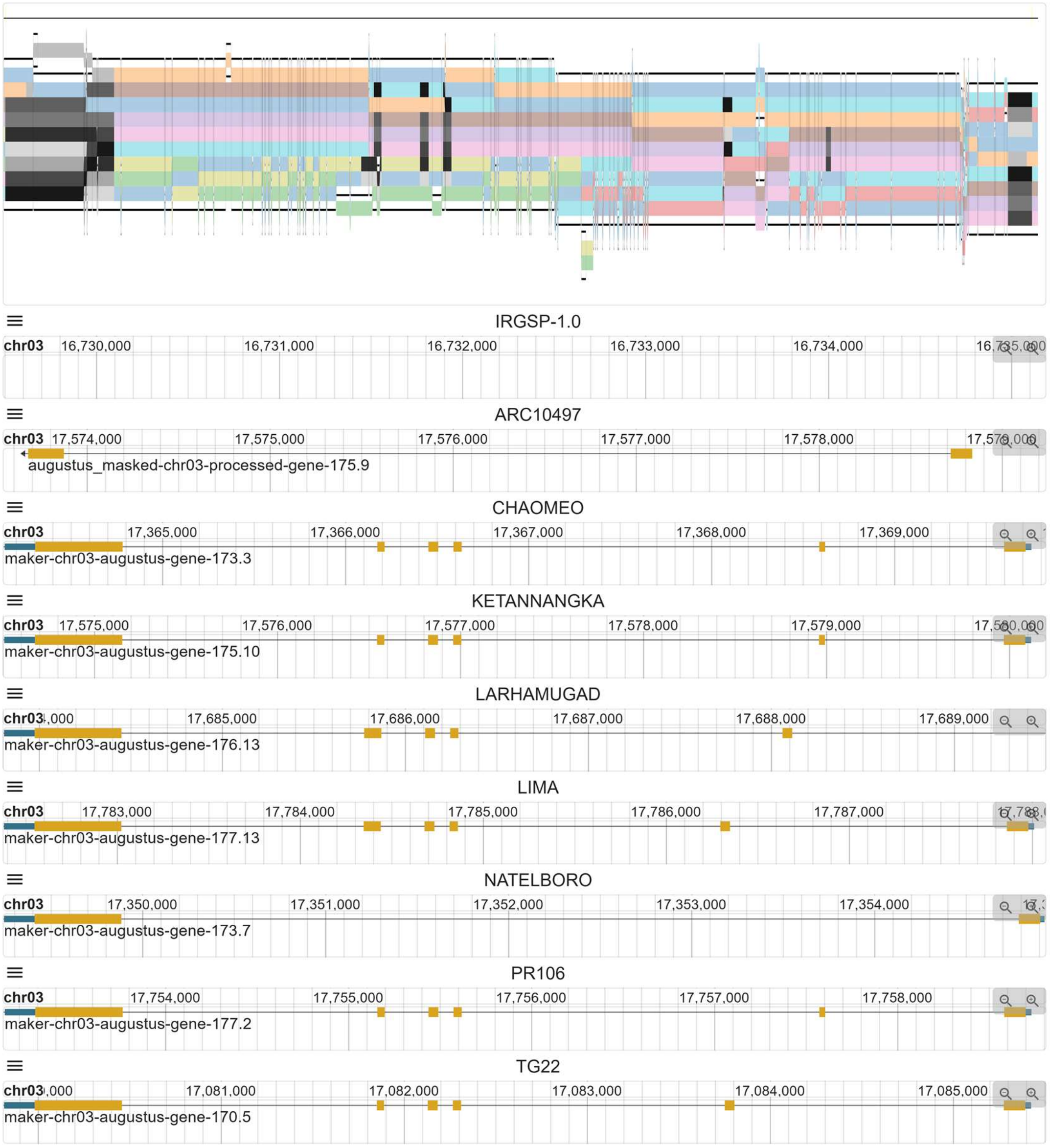

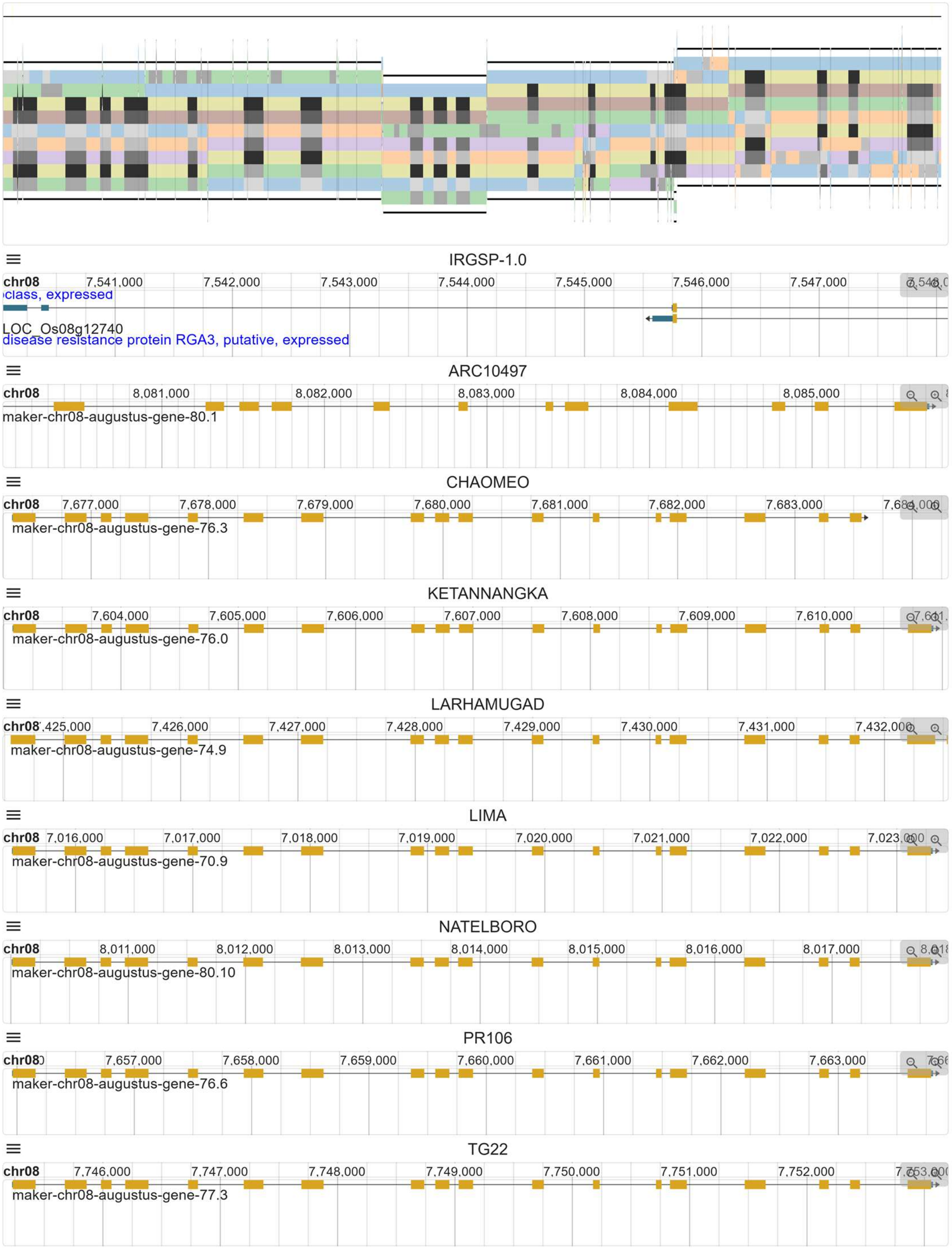
IRGSP-1.0
chr08
7,541,000
7,542,000
7,543,000
7,544,000
7,545,000
7,546,000
7,547,000
class, expressed
LOC_Os08g12740
disease resistance protein RGA3, putative, expressed
ARC10497
chr08
8,081,000
8,082,000
8,083,000
8,084,000
8,085,000
maker-chr08-augustus-gene-80.1
CHAOMEO
chr08
7,677,000
7,678,000
7,679,000
7,680,000
7,681,000
7,682,000
7,683,000
maker-chr08-augustus-gene-76.3
KETANNANGKA
chr08
7,604,000
7,605,000
7,606,000
7,607,000
7,608,000
7,609,000
7,610,000
maker-chr08-augustus-gene-76.0
LARHAMUGAD
chr08
7,426,000
7,427,000
7,428,000
7,429,000
7,430,000
7,431,000
maker-chr08-augustus-gene-74.9
LIMA
chr08
7,016,000
7,017,000
7,018,000
7,019,000
7,020,000
7,021,000
7,022,000
maker-chr08-augustus-gene-70.9
NATELBORO
chr08
8,011,000
8,012,000
8,013,000
8,014,000
8,015,000
8,016,000
8,017,000
maker-chr08-augustus-gene-80.10
PR106
chr08
7,657,000
7,658,000
7,659,000
7,660,000
7,661,000
7,662,000
7,663,000
maker-chr08-augustus-gene-76.6
TG22
chr08
7,746,000
7,747,000
7,748,000
7,749,000
7,750,000
7,751,000
7,752,000
maker-chr08-augustus-gene-77.3

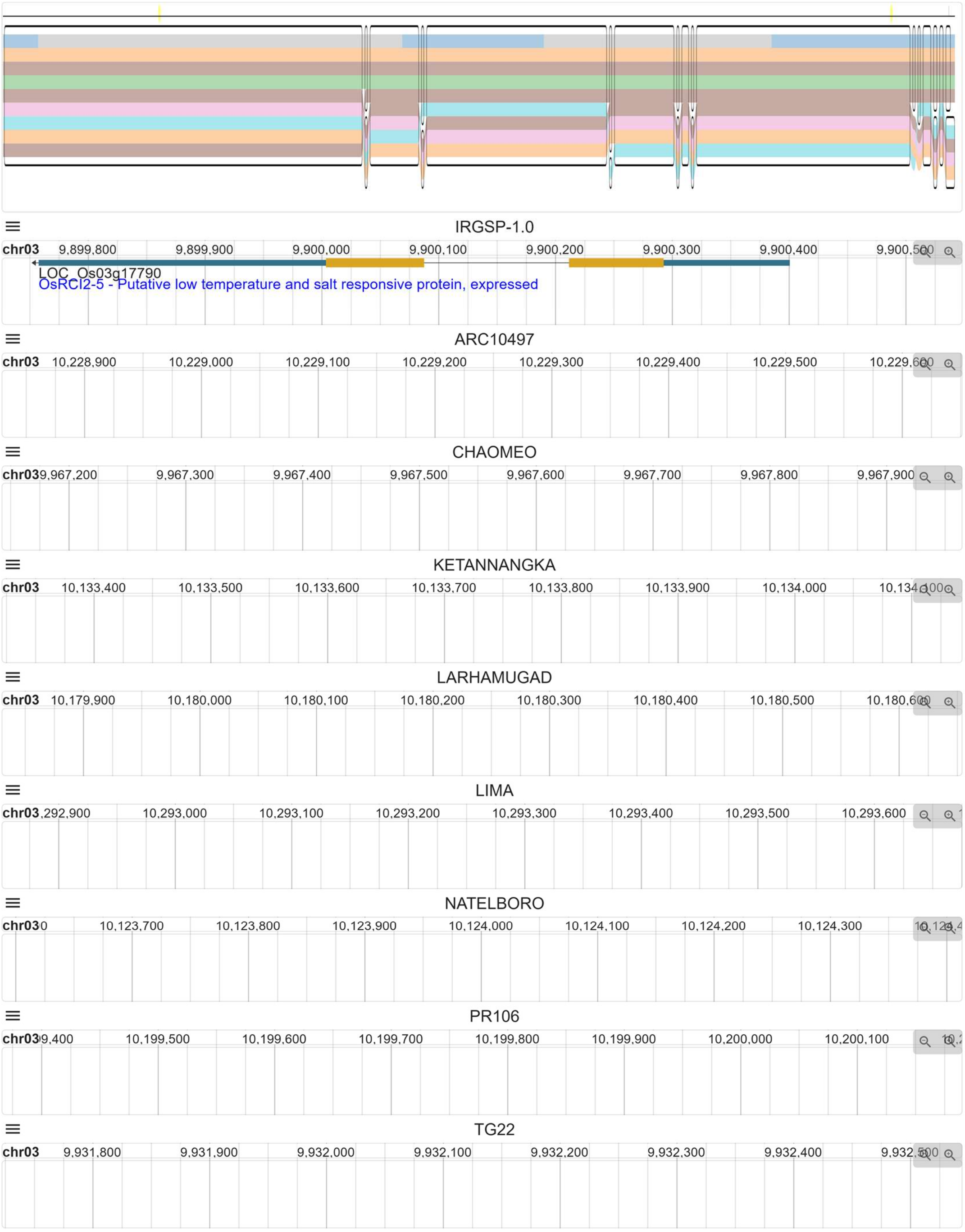
IRGSP-1.0
chr03 9,899,800 9,899,900 9,900,000 9,900,100 9,900,200 9,900,300 9,900,400
LOC_Os03g17790
OsRCI2-5 - Putative low temperature and salt responsive protein, expressed
ARC10497
chr03 10,228,900 10,229,000 10,229,100 10,229,200 10,229,300 10,229,400 10,229,500
CHAOMEO
chr03 9,967,200 9,967,300 9,967,400 9,967,500 9,967,600 9,967,700 9,967,800 9,967,900
KETANNANGKA
chr03 10,133,400 10,133,500 10,133,600 10,133,700 10,133,800 10,133,900 10,134,000
LARHAMUGAD
chr03 10,179,900 10,180,000 10,180,100 10,180,200 10,180,300 10,180,400 10,180,500
LIMA
chr03 10,293,000 10,293,100 10,293,200 10,293,300 10,293,400 10,293,500 10,293,600
NATELBORO
chr03 10,123,700 10,123,800 10,123,900 10,124,000 10,124,100 10,124,200 10,124,300
PR106
chr03 10,199,500 10,199,600 10,199,700 10,199,800 10,199,900 10,200,000 10,200,100
TG22
chr03 9,931,800 9,931,900 9,932,000 9,932,100 9,932,200 9,932,300 9,932,400

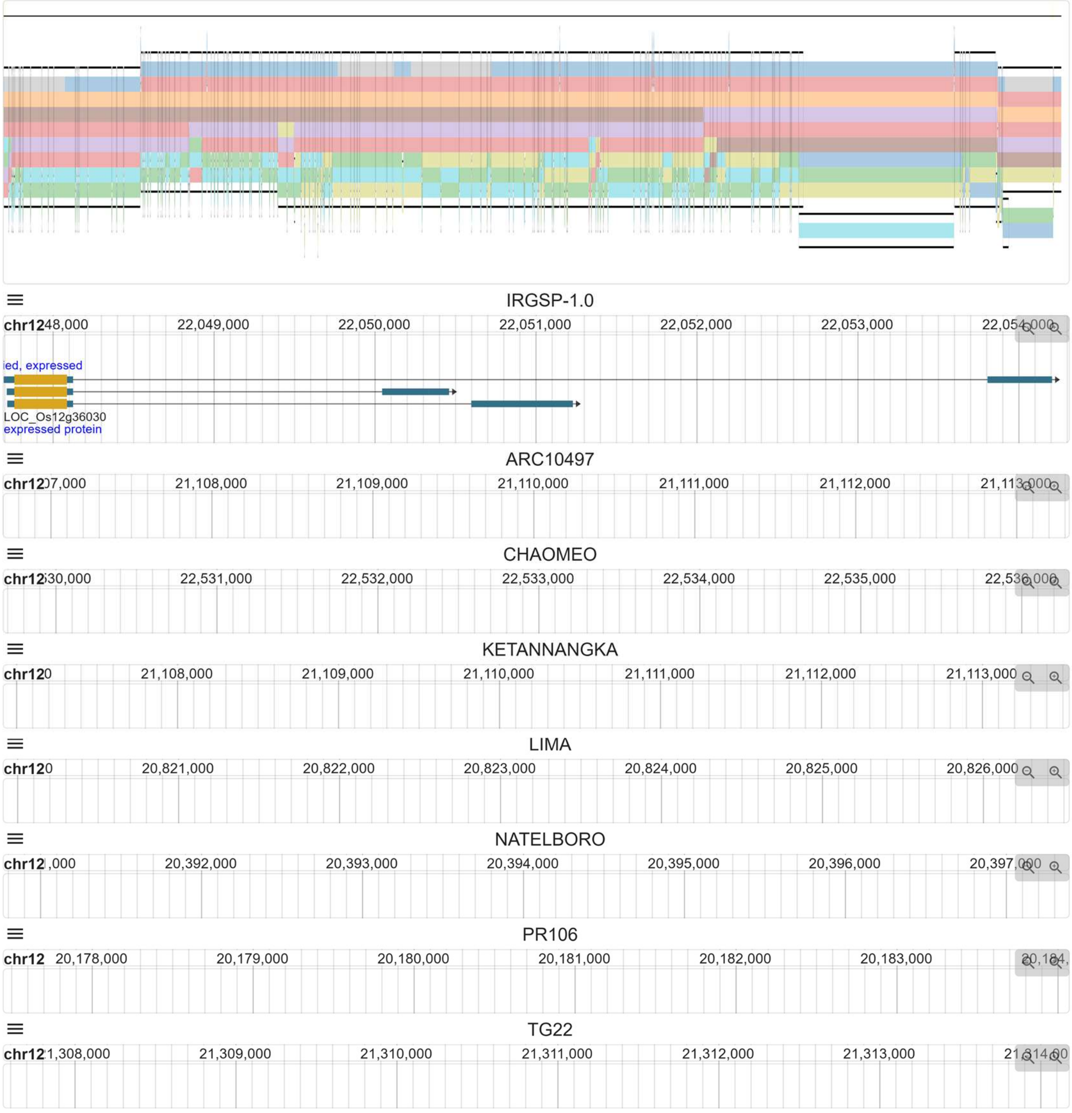
IRGSP-1.0
chr12 48,000
22,049,000
22,050,000
22,051,000
22,052,000
22,053,000
22,054,000
ied, expressed
LOC_Os12g36030
expressed protein
ARC10497
chr12 07,000
21,108,000
21,109,000
21,110,000
21,111,000
21,112,000
21,113,000
CHAOMEO
chr12 30,000
22,531,000
22,532,000
22,533,000
22,534,000
22,535,000
22,536,000
KETANNANGKA
chr12 0
21,108,000
21,109,000
21,110,000
21,111,000
21,112,000
21,113,000
LIMA
chr12 0
20,821,000
20,822,000
20,823,000
20,824,000
20,825,000
20,826,000
NATELBORO
chr12 ,000
20,392,000
20,393,000
20,394,000
20,395,000
20,396,000
20,397,000
PR106
chr12
20,178,000
20,179,000
20,180,000
20,181,000
20,182,000
20,183,000
TG22
chr12 1,308,000
21,309,000
21,310,000
21,311,000
21,312,000
21,313,000
