# Supplementary material for: PPanG: a precision pangenome browser enabling nucleotide-level analysis of genomic variations in individual genomes and their graph-based pangenome
Source: BMC Genomics. 2024 Apr 24;25:405. doi: 10.1186/s12864-024-10302-5 (PMC11044437; doi:10.1186/s12864-024-10302-5)
Supplement: Supplementary file 5 — Supplementary Material 5 [file 12864_2024_10302_MOESM5_ESM.pdf]

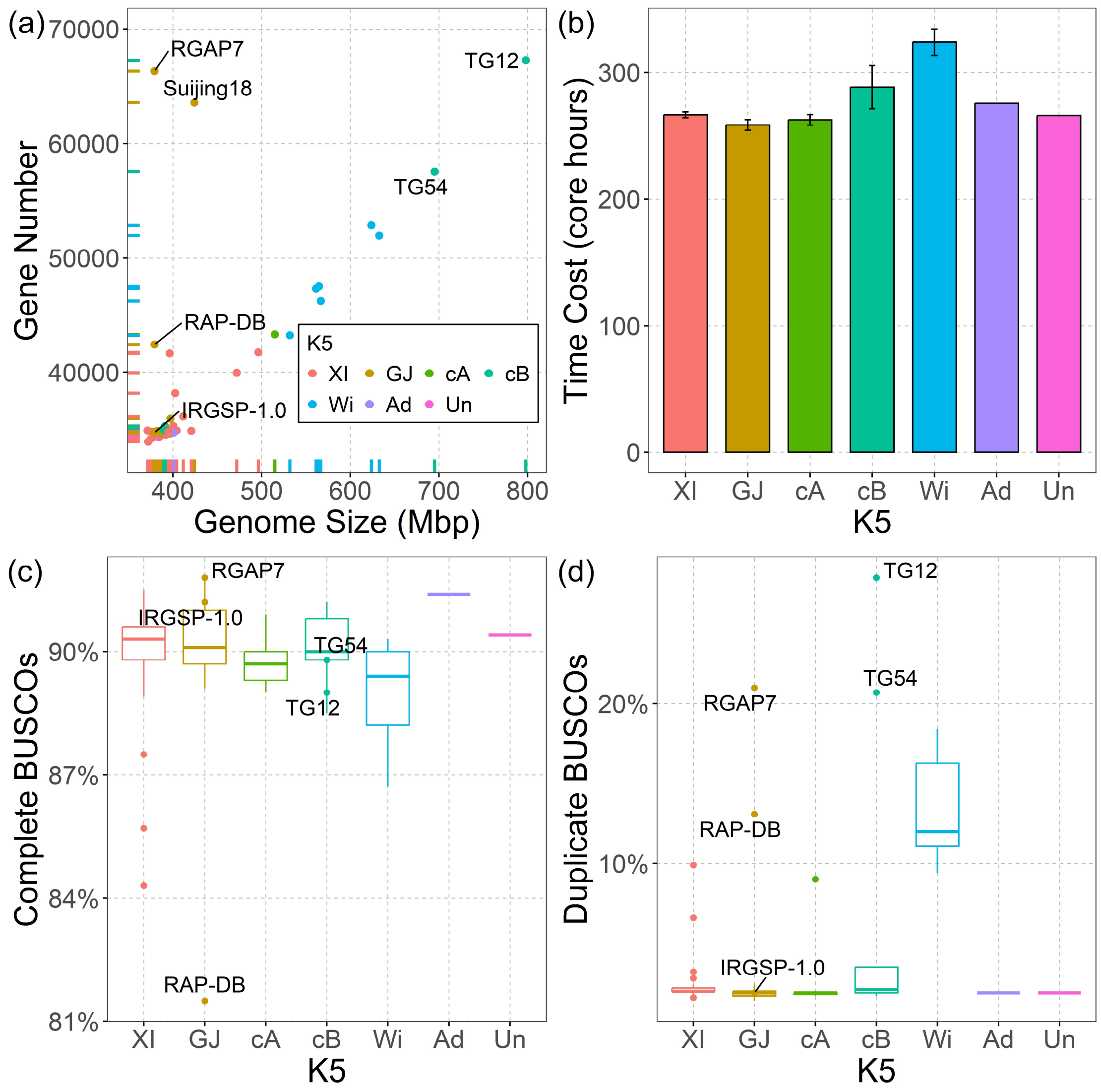

Fig.S1 Statistics about the annotations of 114 rice accessions produced by MAKER. All accessions are grouped by K5 category (GJ: Geng/Japonica, Xi: Xian/Indica, cA: circum-Aus, cB: circum-Basmati, Wi: Wild, Ad: Admix, Un: Unknown). Some significant outliers and reference genome IRGSP-1.0 are marked in labels. Existing reference genome annotations RAP-DB (International Rice Genome Sequencing Project, Rice Annotation Project Database, version 1.0, RAP-DB/2021-11-11, https://rapdb.dna.affrc.go.jp/download/irgsp1.html) and RGAP7 (Michigan State University Rice Genome Annotation Project Release 7, MSU RGAP7, http://rice.uga.edu/downloads.shtml) are also labelled for comparison. **a)** The genome size and annotated gene number of each individual. **b)** The running time (in core hours) for MAKER annotations. **c)** The score of complete BUSCOs evaluated at

protein level. **d)** The score of complete and duplicated BUSCOs evaluated at protein level.
