# Supplementary material for: PPanG: a precision pangenome browser enabling nucleotide-level analysis of genomic variations in individual genomes and their graph-based pangenome
Source: BMC Genomics. 2024 Apr 24;25:405. doi: 10.1186/s12864-024-10302-5 (PMC11044437; doi:10.1186/s12864-024-10302-5)

Fig.S3 Comparison of special regions from pangenome graphs built by MC and PGGB. **a)** Region: IRGSP-1.0.chr05: 5366417-5366577 **b)** Region: IRGSP-1.0.chr06: 28873798-28873958

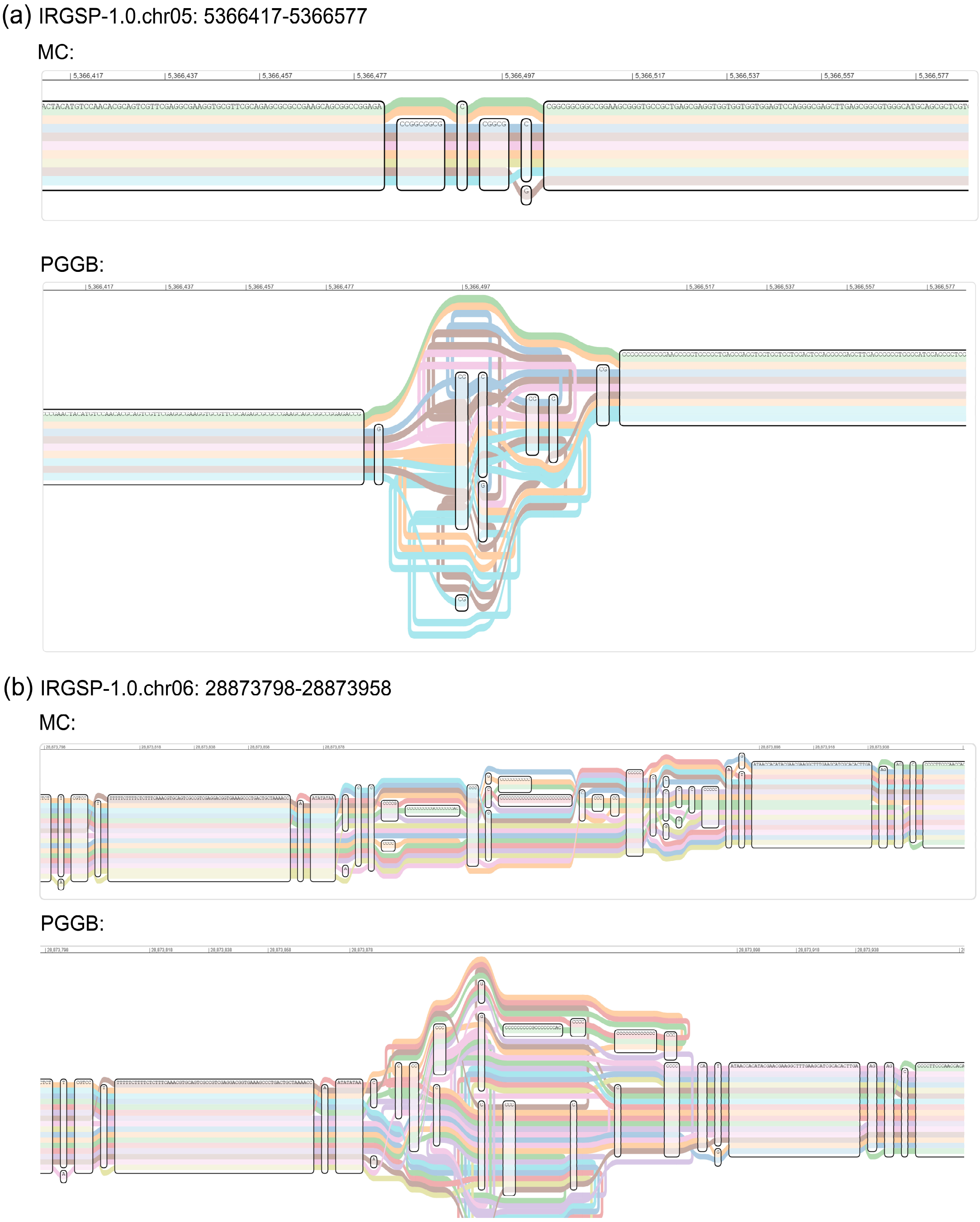

Supplement: Supplementary file 6 — Supplementary Material 6 [file 12864_2024_10302_MOESM6_ESM.pdf]
