# Supplementary material for: PPanG: a precision pangenome browser enabling nucleotide-level analysis of genomic variations in individual genomes and their graph-based pangenome
Source: BMC Genomics. 2024 Apr 24;25:405. doi: 10.1186/s12864-024-10302-5 (PMC11044437; doi:10.1186/s12864-024-10302-5)
Supplement: Supplementary file 7 — Supplementary Material 7 [file 12864_2024_10302_MOESM7_ESM.pdf]

Fig.S2 Diagnostic visualizations of 12 pangenome graphs provided by PGGB. These diagnostic figures indicate high synteny in 113 rice samples.

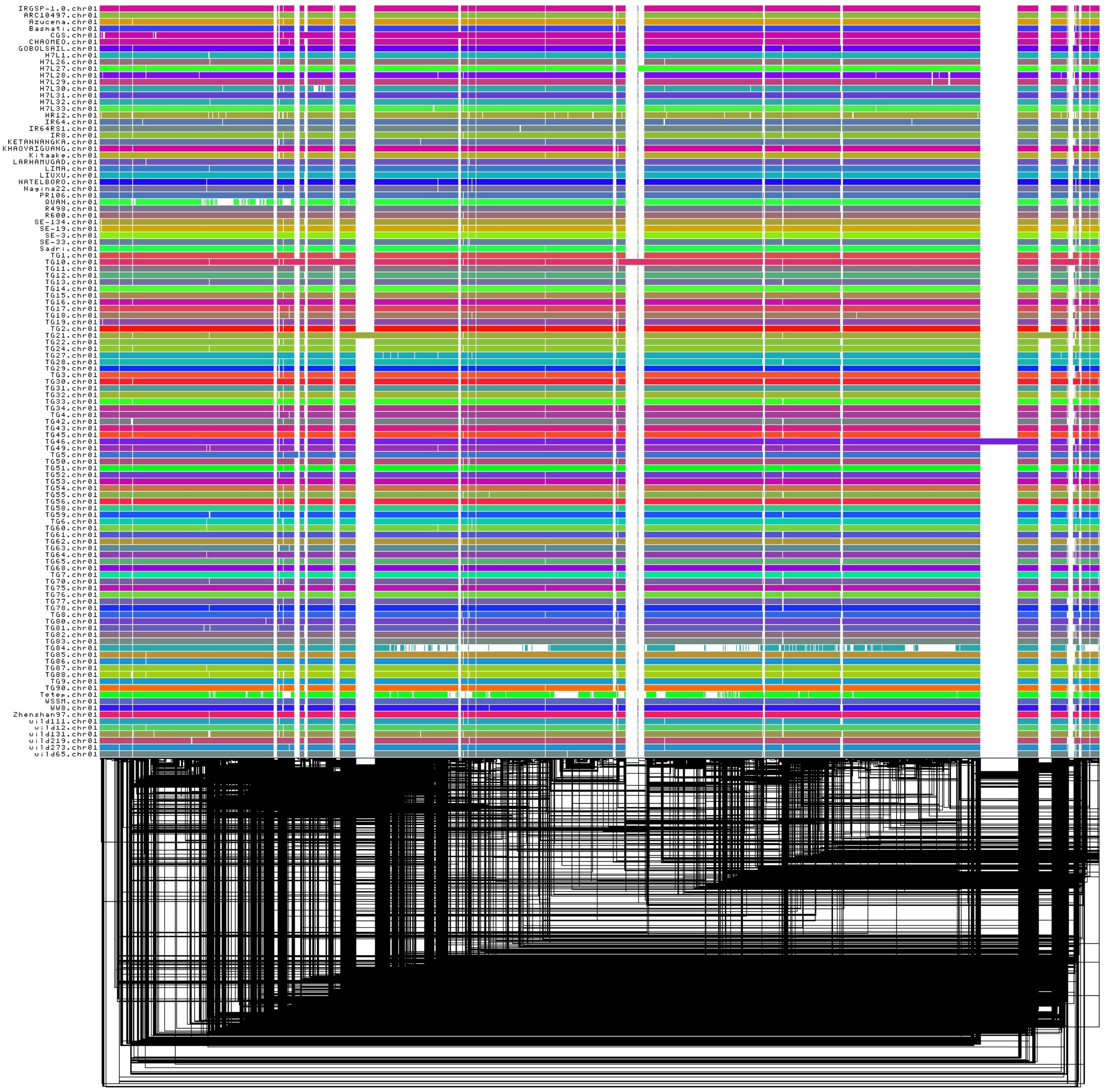

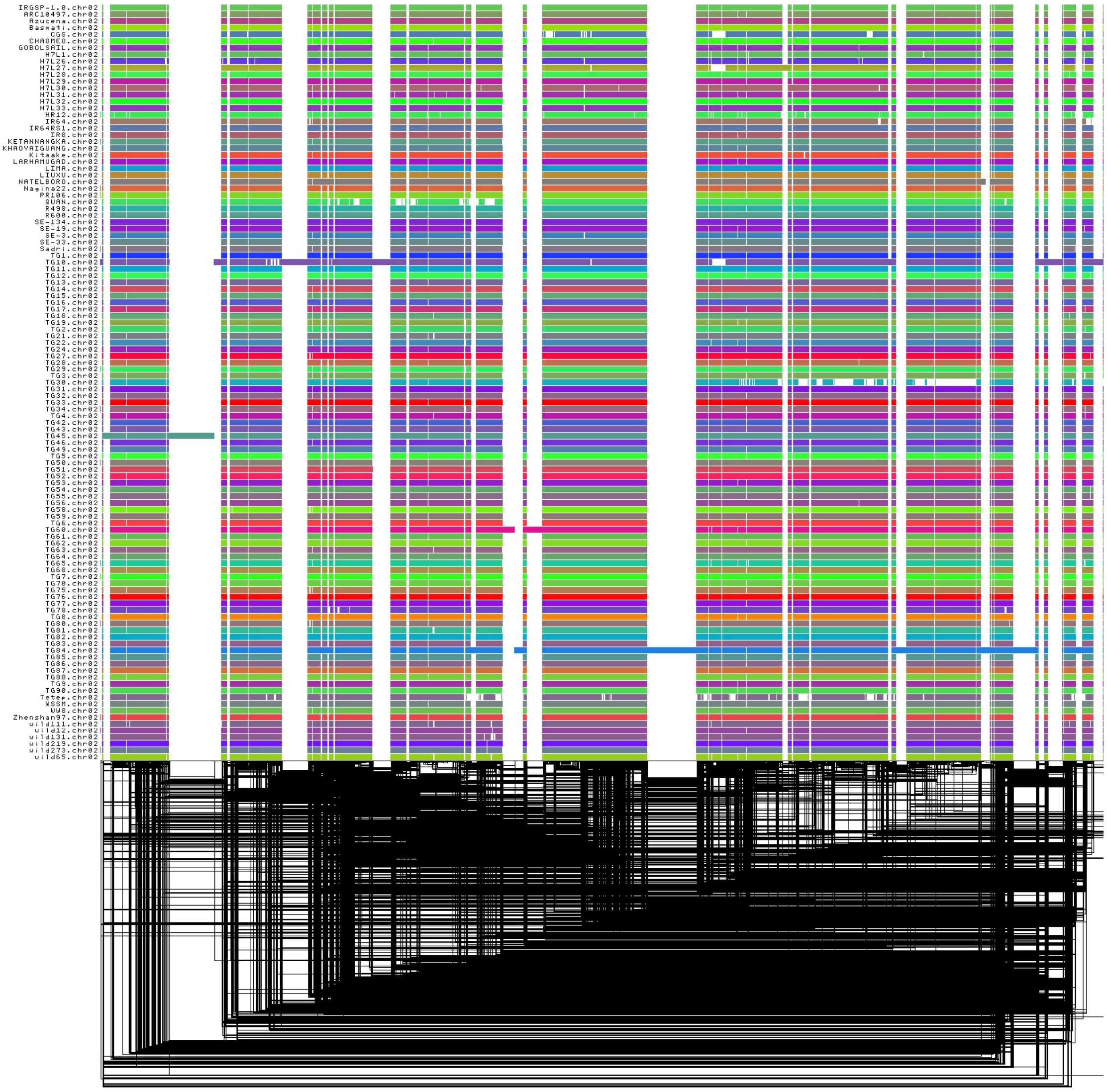

IRGSP-1.0.chr02
ARC10497.chr02
Azucena.chr02
Basmati.chr02
CGS.chr02
CHAOMEO.chr02
GOBOLSAIL.chr02
H7L1.chr02
H7L26.chr02
H7L27.chr02
H7L28.chr02
H7L29.chr02
H7L30.chr02
H7L31.chr02
H7L32.chr02
H7L33.chr02
HR12.chr02
IR64.chr02
IR64RS1.chr02
IR8.chr02
KETANNANGKA.chr02
KHAOYAIGUANG.chr02
Kitaake.chr02
LARHAMUGAD.chr02
LIMA.chr02
LIUXU.chr02
NATELBORO.chr02
Nagina22.chr02
PR106.chr02
QUAN.chr02
R498.chr02
R600.chr02
SE-134.chr02
SE-19.chr02
SE-3.chr02
SE-33.chr02
Sadri.chr02
TG1.chr02
TG10.chr02
TG11.chr02
TG12.chr02
TG13.chr02
TG14.chr02
TG15.chr02
TG16.chr02
TG17.chr02
TG18.chr02
TG19.chr02
TG2.chr02
TG21.chr02
TG22.chr02
TG24.chr02
TG27.chr02
TG28.chr02
TG29.chr02
TG3.chr02
TG30.chr02
TG31.chr02
TG32.chr02
TG33.chr02
TG34.chr02
TG4.chr02
TG42.chr02
TG43.chr02
TG45.chr02
TG46.chr02
TG49.chr02
TG5.chr02
TG50.chr02
TG51.chr02
TG52.chr02
TG53.chr02
TG54.chr02
TG55.chr02
TG56.chr02
TG58.chr02
TG59.chr02
TG6.chr02
TG60.chr02
TG61.chr02
TG62.chr02
TG63.chr02
TG64.chr02
TG65.chr02
TG68.chr02
TG7.chr02
TG70.chr02
TG75.chr02
TG76.chr02
TG77.chr02
TG78.chr02
TG8.chr02
TG80.chr02
TG81.chr02
TG82.chr02
TG83.chr02
TG84.chr02
TG85.chr02
TG86.chr02
TG87.chr02
TG88.chr02
TG9.chr02
TG90.chr02
Tetep.chr02
WSSM.chr02
WW8.chr02
Zhenshan97.chr02
wild111.chr02
wild12.chr02
wild131.chr02
wild219.chr02
wild273.chr02
wild65.chr02

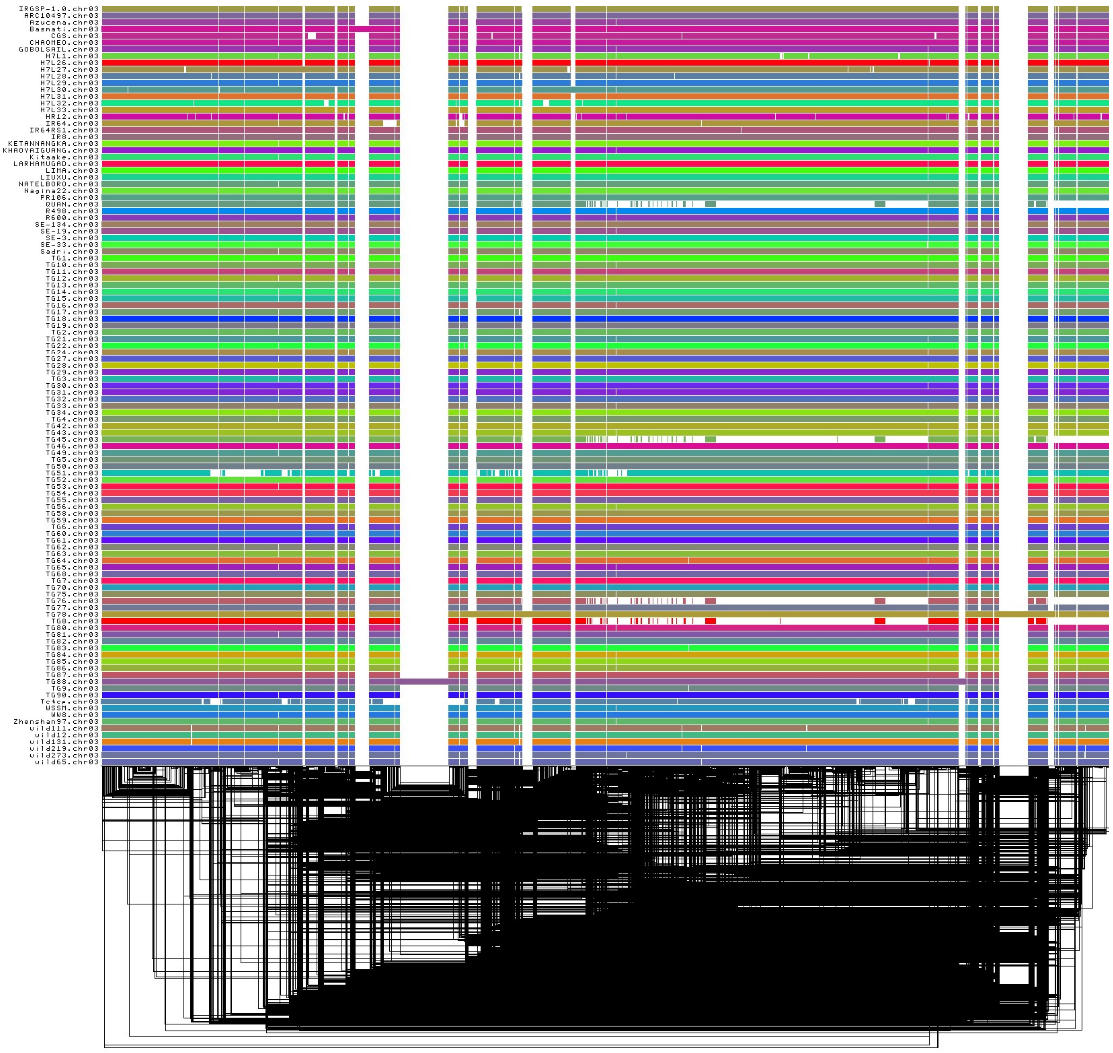

IRGSP-1.0.chr03
ARC10497.chr03
Azucena.chr03
Basmati.chr03
CGS.chr03
CHAOMEO.chr03
GOBOLSAIL.chr03
H7L1.chr03
H7L26.chr03
H7L27.chr03
H7L28.chr03
H7L29.chr03
H7L30.chr03
H7L31.chr03
H7L32.chr03
H7L33.chr03
HR12.chr03
IR64.chr03
IR64RS1.chr03
IR8.chr03
KETANNANGKA.chr03
KHAOYAIGUANG.chr03
Kitaake.chr03
LARHAMUGAD.chr03
LIMA.chr03
LIUXU.chr03
NATELBORO.chr03
Nagina22.chr03
PR106.chr03
QUAN.chr03
R498.chr03
R600.chr03
SE-134.chr03
SE-19.chr03
SE-3.chr03
SE-33.chr03
Sadri.chr03
TG1.chr03
TG10.chr03
TG11.chr03
TG12.chr03
TG13.chr03
TG14.chr03
TG15.chr03
TG16.chr03
TG17.chr03
TG18.chr03
TG19.chr03
TG2.chr03
TG21.chr03
TG22.chr03
TG24.chr03
TG27.chr03
TG28.chr03
TG29.chr03
TG3.chr03
TG30.chr03
TG31.chr03
TG32.chr03
TG33.chr03
TG34.chr03
TG4.chr03
TG42.chr03
TG43.chr03
TG45.chr03
TG46.chr03
TG49.chr03
TG5.chr03
TG50.chr03
TG51.chr03
TG52.chr03
TG53.chr03
TG54.chr03
TG55.chr03
TG56.chr03
TG58.chr03
TG59.chr03
TG6.chr03
TG60.chr03
TG61.chr03
TG62.chr03
TG63.chr03
TG64.chr03
TG65.chr03
TG68.chr03
TG7.chr03
TG70.chr03
TG75.chr03
TG76.chr03
TG77.chr03
TG78.chr03
TG8.chr03
TG80.chr03
TG81.chr03
TG82.chr03
TG83.chr03
TG84.chr03
TG85.chr03
TG86.chr03
TG87.chr03
TG88.chr03
TG9.chr03
TG90.chr03
Tetep.chr03
WSSM.chr03
WW8.chr03
Zhenshan97.chr03
wild111.chr03
wild12.chr03
wild131.chr03
wild219.chr03
wild273.chr03
wild65.chr03

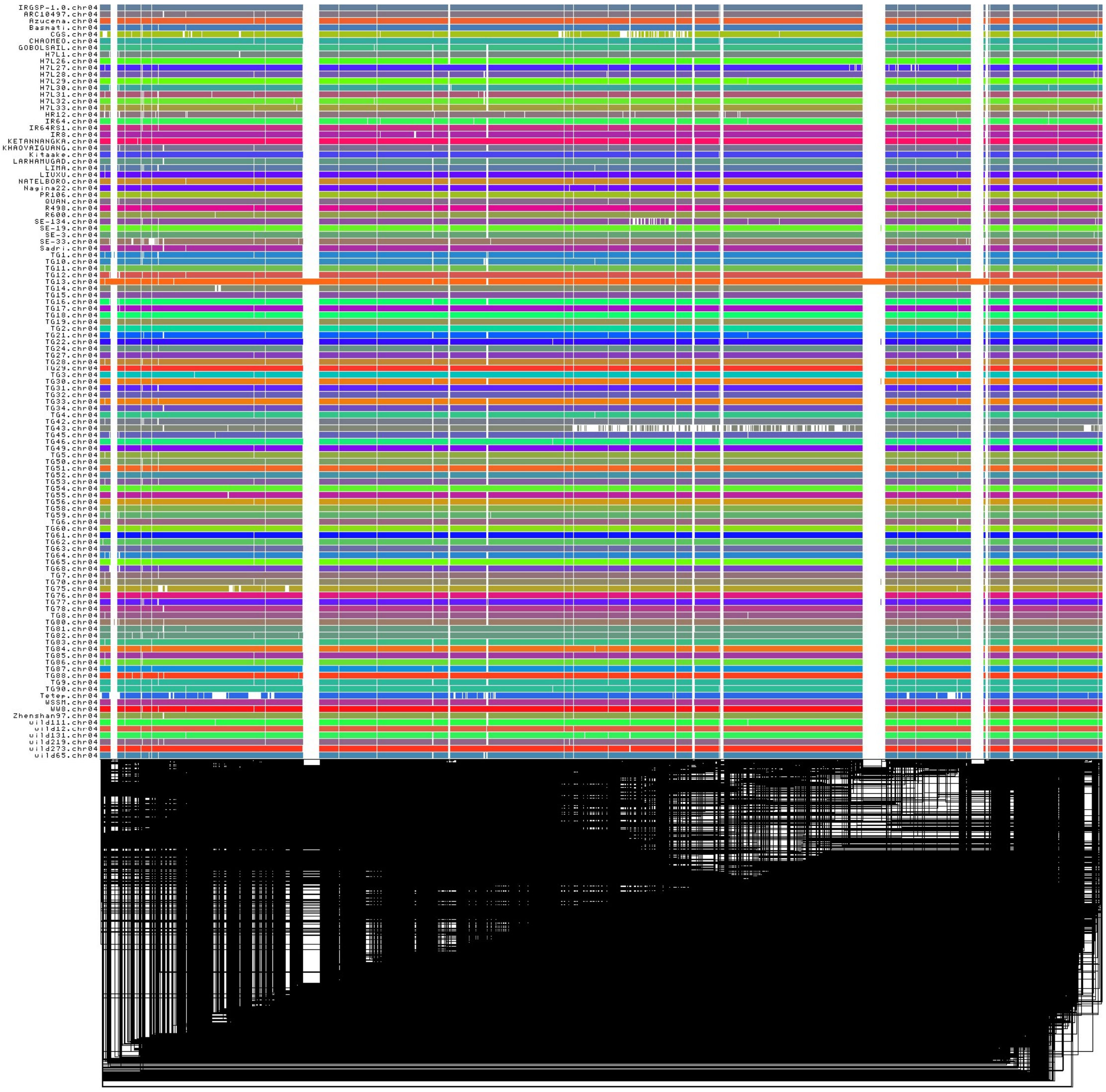
IRGSP-1.0.chr04
ARC10497.chr04
Azucena.chr04
Basmati.chr04
CGS.chr04
CHAOMEO.chr04
GOBOLSAIL.chr04
H7L1.chr04
H7L26.chr04
H7L27.chr04
H7L28.chr04
H7L29.chr04
H7L30.chr04
H7L31.chr04
H7L32.chr04
H7L33.chr04
HR12.chr04
IR64.chr04
IR64RS1.chr04
IR8.chr04
KETANNANGKA.chr04
KHAOYAIGUANG.chr04
Kitaake.chr04
LARHAMUGAD.chr04
LIMA.chr04
LIUXU.chr04
NATELBORO.chr04
Nagina22.chr04
PR106.chr04
QUAN.chr04
R498.chr04
R600.chr04
SE-134.chr04
SE-19.chr04
SE-3.chr04
SE-33.chr04
Sadri.chr04
TG1.chr04
TG10.chr04
TG11.chr04
TG12.chr04
TG13.chr04
TG14.chr04
TG15.chr04
TG16.chr04
TG17.chr04
TG18.chr04
TG19.chr04
TG2.chr04
TG21.chr04
TG22.chr04
TG24.chr04
TG27.chr04
TG28.chr04
TG29.chr04
TG3.chr04
TG30.chr04
TG31.chr04
TG32.chr04
TG33.chr04
TG34.chr04
TG4.chr04
TG42.chr04
TG43.chr04
TG45.chr04
TG46.chr04
TG49.chr04
TG5.chr04
TG50.chr04
TG51.chr04
TG52.chr04
TG53.chr04
TG54.chr04
TG55.chr04
TG56.chr04
TG58.chr04
TG59.chr04
TG6.chr04
TG60.chr04
TG61.chr04
TG62.chr04
TG63.chr04
TG64.chr04
TG65.chr04
TG68.chr04
TG7.chr04
TG70.chr04
TG75.chr04
TG76.chr04
TG77.chr04
TG78.chr04
TG8.chr04
TG80.chr04
TG81.chr04
TG82.chr04
TG83.chr04
TG84.chr04
TG85.chr04
TG86.chr04
TG87.chr04
TG88.chr04
TG9.chr04
TG90.chr04
Tetep.chr04
WSSM.chr04
WW8.chr04
Zhenshan97.chr04
wild111.chr04
wild12.chr04
wild131.chr04
wild219.chr04
wild273.chr04
wild65.chr04

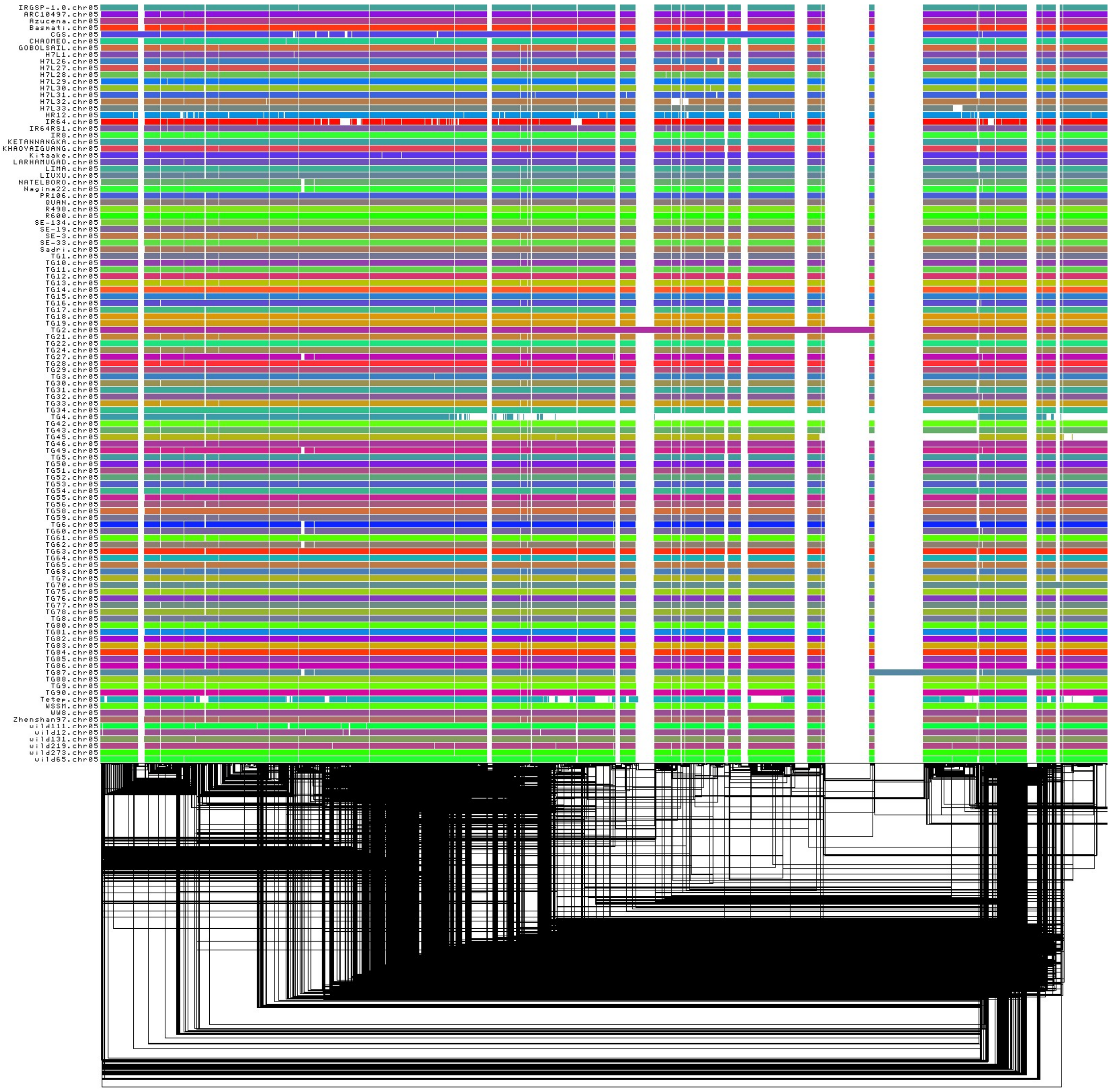
IRGSP-1.0.chr05
ARC10497.chr05
Azucena.chr05
Basmati.chr05
CGS.chr05
CHAOMEO.chr05
GOBOLSAIL.chr05
H7L1.chr05
H7L26.chr05
H7L27.chr05
H7L28.chr05
H7L29.chr05
H7L30.chr05
H7L31.chr05
H7L32.chr05
H7L33.chr05
HR12.chr05
IR64.chr05
IR64RS1.chr05
IR8.chr05
KETANNANGKA.chr05
KHAOYAIGUANG.chr05
Kitaake.chr05
LARHAMUGAD.chr05
LIMA.chr05
LIUXU.chr05
NATELBORO.chr05
Nagina22.chr05
PR106.chr05
QUAN.chr05
R498.chr05
R600.chr05
SE-134.chr05
SE-19.chr05
SE-3.chr05
SE-33.chr05
Sadri.chr05
TG1.chr05
TG10.chr05
TG11.chr05
TG12.chr05
TG13.chr05
TG14.chr05
TG15.chr05
TG16.chr05
TG17.chr05
TG18.chr05
TG19.chr05
TG2.chr05
TG21.chr05
TG22.chr05
TG24.chr05
TG27.chr05
TG28.chr05
TG29.chr05
TG3.chr05
TG30.chr05
TG31.chr05
TG32.chr05
TG33.chr05
TG34.chr05
TG4.chr05
TG42.chr05
TG43.chr05
TG45.chr05
TG46.chr05
TG49.chr05
TG5.chr05
TG50.chr05
TG51.chr05
TG52.chr05
TG53.chr05
TG54.chr05
TG55.chr05
TG56.chr05
TG58.chr05
TG59.chr05
TG6.chr05
TG60.chr05
TG61.chr05
TG62.chr05
TG63.chr05
TG64.chr05
TG65.chr05
TG68.chr05
TG7.chr05
TG70.chr05
TG75.chr05
TG76.chr05
TG77.chr05
TG78.chr05
TG8.chr05
TG80.chr05
TG81.chr05
TG82.chr05
TG83.chr05
TG84.chr05
TG85.chr05
TG86.chr05
TG87.chr05
TG88.chr05
TG9.chr05
TG90.chr05
Tetep.chr05
WSSM.chr05
WW8.chr05
Zhenshan97.chr05
wild111.chr05
wild12.chr05
wild131.chr05
wild219.chr05
wild273.chr05
wild65.chr05

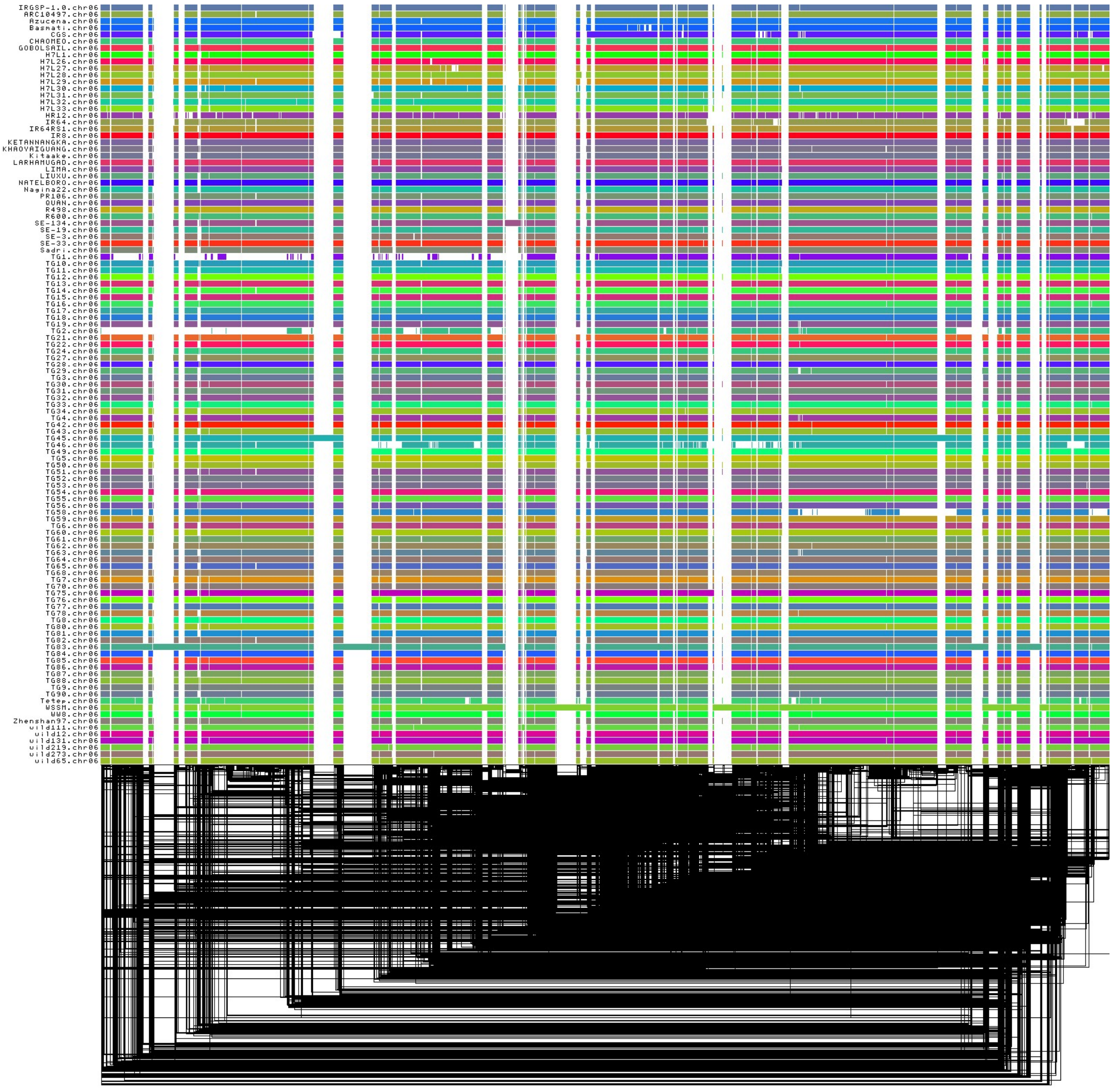
IRGSP-1.0.chr06
ARC10497.chr06
Azucena.chr06
Basmati.chr06
CGS.chr06
CHAOMEO.chr06
GOBOLSAIL.chr06
H7L1.chr06
H7L26.chr06
H7L27.chr06
H7L28.chr06
H7L29.chr06
H7L30.chr06
H7L31.chr06
H7L32.chr06
H7L33.chr06
HR12.chr06
IR64.chr06
IR64RS1.chr06
IR8.chr06
KETANNANGKA.chr06
KHAOYAIGUANG.chr06
Kitaake.chr06
LARHAMUGAD.chr06
LIMA.chr06
LIUXU.chr06
NATELBORO.chr06
Nagina22.chr06
PR106.chr06
QUAN.chr06
R498.chr06
R600.chr06
SE-134.chr06
SE-19.chr06
SE-3.chr06
SE-33.chr06
Sadri.chr06
TG1.chr06
TG10.chr06
TG11.chr06
TG12.chr06
TG13.chr06
TG14.chr06
TG15.chr06
TG16.chr06
TG17.chr06
TG18.chr06
TG19.chr06
TG2.chr06
TG21.chr06
TG22.chr06
TG24.chr06
TG27.chr06
TG28.chr06
TG29.chr06
TG3.chr06
TG30.chr06
TG31.chr06
TG32.chr06
TG33.chr06
TG34.chr06
TG4.chr06
TG42.chr06
TG43.chr06
TG45.chr06
TG46.chr06
TG49.chr06
TG5.chr06
TG50.chr06
TG51.chr06
TG52.chr06
TG53.chr06
TG54.chr06
TG55.chr06
TG56.chr06
TG58.chr06
TG59.chr06
TG6.chr06
TG60.chr06
TG61.chr06
TG62.chr06
TG63.chr06
TG64.chr06
TG65.chr06
TG68.chr06
TG7.chr06
TG70.chr06
TG75.chr06
TG76.chr06
TG77.chr06
TG78.chr06
TG8.chr06
TG80.chr06
TG81.chr06
TG82.chr06
TG83.chr06
TG84.chr06
TG85.chr06
TG86.chr06
TG87.chr06
TG88.chr06
TG9.chr06
TG90.chr06
Tetep.chr06
WSSM.chr06
WW8.chr06
Zhenshan97.chr06
wild111.chr06
wild12.chr06
wild131.chr06
wild219.chr06
wild273.chr06
wild65.chr06

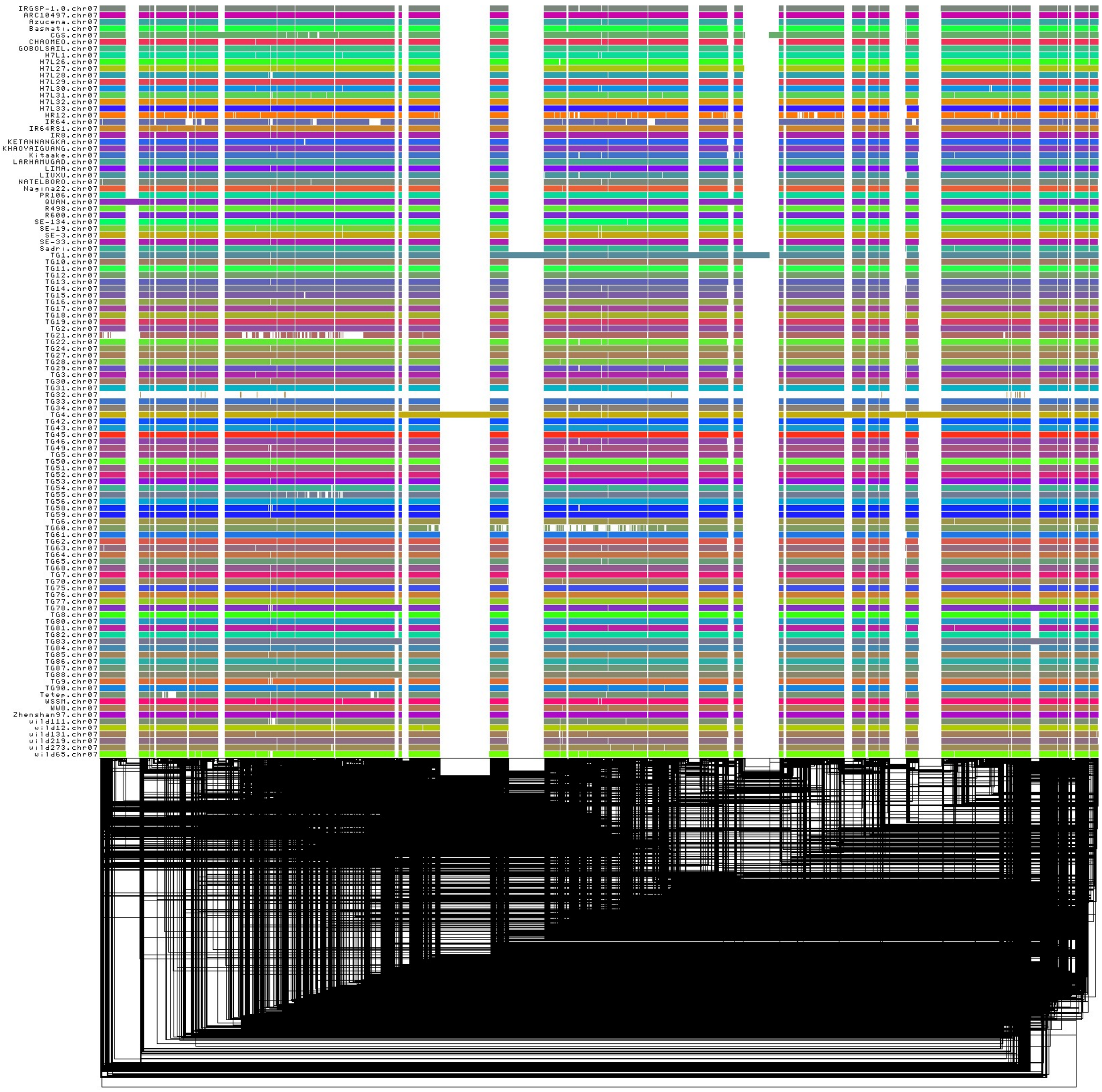
IRGSP-1.0.chr07
ARC10497.chr07
Azucena.chr07
Basmati.chr07
CGS.chr07
CHAOMEO.chr07
GOBOLSAIL.chr07
H7L1.chr07
H7L26.chr07
H7L27.chr07
H7L28.chr07
H7L29.chr07
H7L30.chr07
H7L31.chr07
H7L32.chr07
H7L33.chr07
HR12.chr07
IR64.chr07
IR64RS1.chr07
IR8.chr07
KETANNANGKA.chr07
KHAOYAIGUANG.chr07
Kitaake.chr07
LARHAMUGAD.chr07
LIMA.chr07
LIUXU.chr07
NATELBORO.chr07
Nagina22.chr07
PR106.chr07
QUAN.chr07
R498.chr07
R600.chr07
SE-134.chr07
SE-19.chr07
SE-3.chr07
SE-33.chr07
Sadri.chr07
TG1.chr07
TG10.chr07
TG11.chr07
TG12.chr07
TG13.chr07
TG14.chr07
TG15.chr07
TG16.chr07
TG17.chr07
TG18.chr07
TG19.chr07
TG2.chr07
TG21.chr07
TG22.chr07
TG24.chr07
TG27.chr07
TG28.chr07
TG29.chr07
TG3.chr07
TG30.chr07
TG31.chr07
TG32.chr07
TG33.chr07
TG34.chr07
TG4.chr07
TG42.chr07
TG43.chr07
TG45.chr07
TG46.chr07
TG49.chr07
TG5.chr07
TG50.chr07
TG51.chr07
TG52.chr07
TG53.chr07
TG54.chr07
TG55.chr07
TG56.chr07
TG58.chr07
TG59.chr07
TG6.chr07
TG60.chr07
TG61.chr07
TG62.chr07
TG63.chr07
TG64.chr07
TG65.chr07
TG68.chr07
TG7.chr07
TG70.chr07
TG75.chr07
TG76.chr07
TG77.chr07
TG78.chr07
TG8.chr07
TG80.chr07
TG81.chr07
TG82.chr07
TG83.chr07
TG84.chr07
TG85.chr07
TG86.chr07
TG87.chr07
TG88.chr07
TG9.chr07
TG90.chr07
Tetep.chr07
WSSM.chr07
WW8.chr07
Zhenshan97.chr07
wild111.chr07
wild12.chr07
wild131.chr07
wild219.chr07
wild273.chr07
wild65.chr07

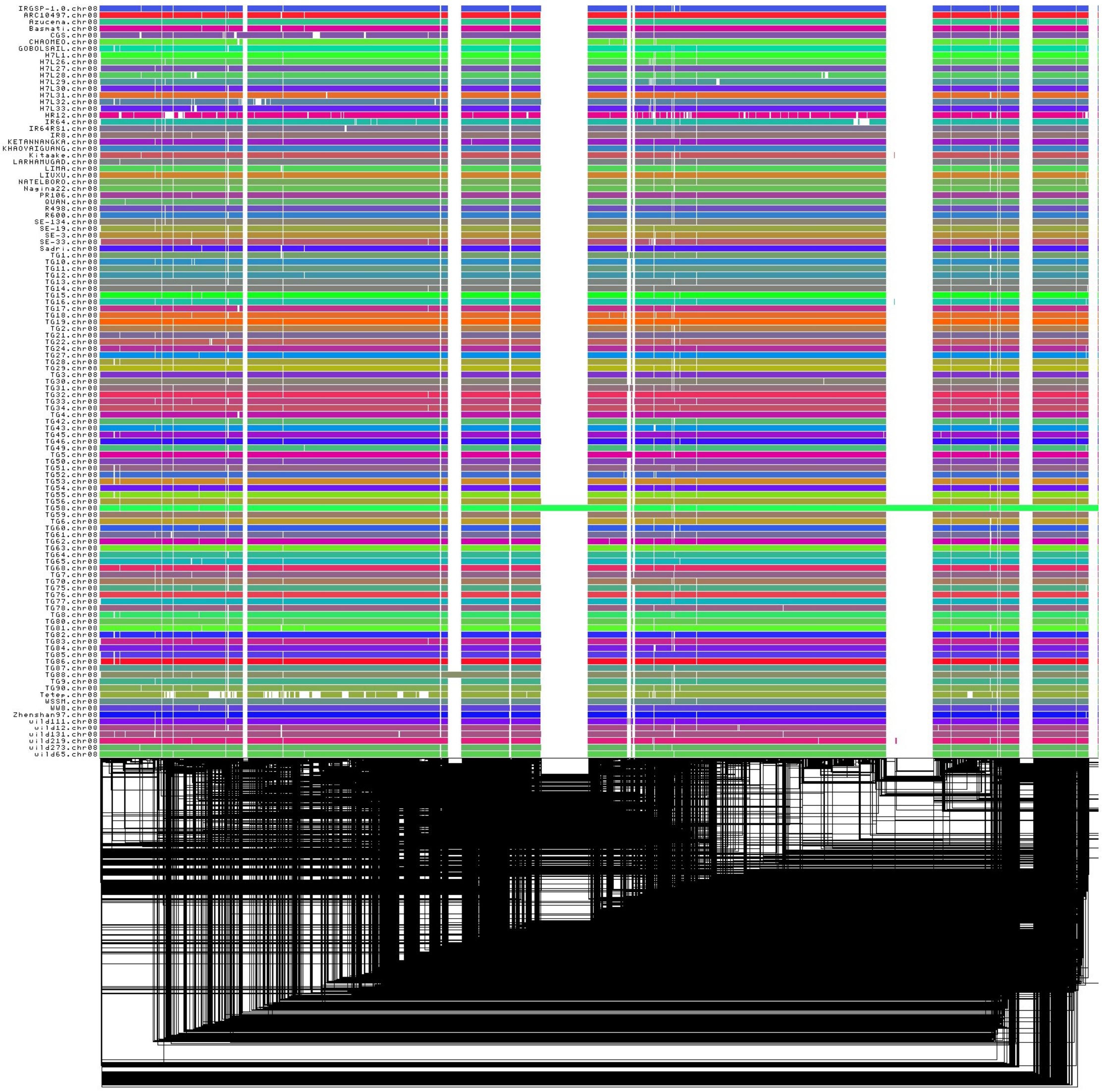

IRGSP-1.0.chr08
ARC10497.chr08
Azucena.chr08
Basmati.chr08
CGS.chr08
CHAOMEO.chr08
GOBOLSAIL.chr08
H7L1.chr08
H7L26.chr08
H7L27.chr08
H7L28.chr08
H7L29.chr08
H7L30.chr08
H7L31.chr08
H7L32.chr08
H7L33.chr08
HR12.chr08
IR64.chr08
IR64RS1.chr08
IR8.chr08
KETANNANGKA.chr08
KHAOYAIGUANG.chr08
Kitaake.chr08
LARHAMUGAD.chr08
LIMA.chr08
LIUXU.chr08
NATELBORO.chr08
Nagina22.chr08
PR106.chr08
QUAN.chr08
R498.chr08
R600.chr08
SE-134.chr08
SE-19.chr08
SE-3.chr08
SE-33.chr08
Sadri.chr08
TG1.chr08
TG10.chr08
TG11.chr08
TG12.chr08
TG13.chr08
TG14.chr08
TG15.chr08
TG16.chr08
TG17.chr08
TG18.chr08
TG19.chr08
TG2.chr08
TG21.chr08
TG22.chr08
TG24.chr08
TG27.chr08
TG28.chr08
TG29.chr08
TG3.chr08
TG30.chr08
TG31.chr08
TG32.chr08
TG33.chr08
TG34.chr08
TG4.chr08
TG42.chr08
TG43.chr08
TG45.chr08
TG46.chr08
TG49.chr08
TG5.chr08
TG50.chr08
TG51.chr08
TG52.chr08
TG53.chr08
TG54.chr08
TG55.chr08
TG56.chr08
TG58.chr08
TG59.chr08
TG6.chr08
TG60.chr08
TG61.chr08
TG62.chr08
TG63.chr08
TG64.chr08
TG65.chr08
TG68.chr08
TG7.chr08
TG70.chr08
TG75.chr08
TG76.chr08
TG77.chr08
TG78.chr08
TG8.chr08
TG80.chr08
TG81.chr08
TG82.chr08
TG83.chr08
TG84.chr08
TG85.chr08
TG86.chr08
TG87.chr08
TG88.chr08
TG9.chr08
TG90.chr08
Tetep.chr08
WSSM.chr08
WW8.chr08
Zhenshan97.chr08
wild111.chr08
wild12.chr08
wild131.chr08
wild219.chr08
wild273.chr08
wild65.chr08

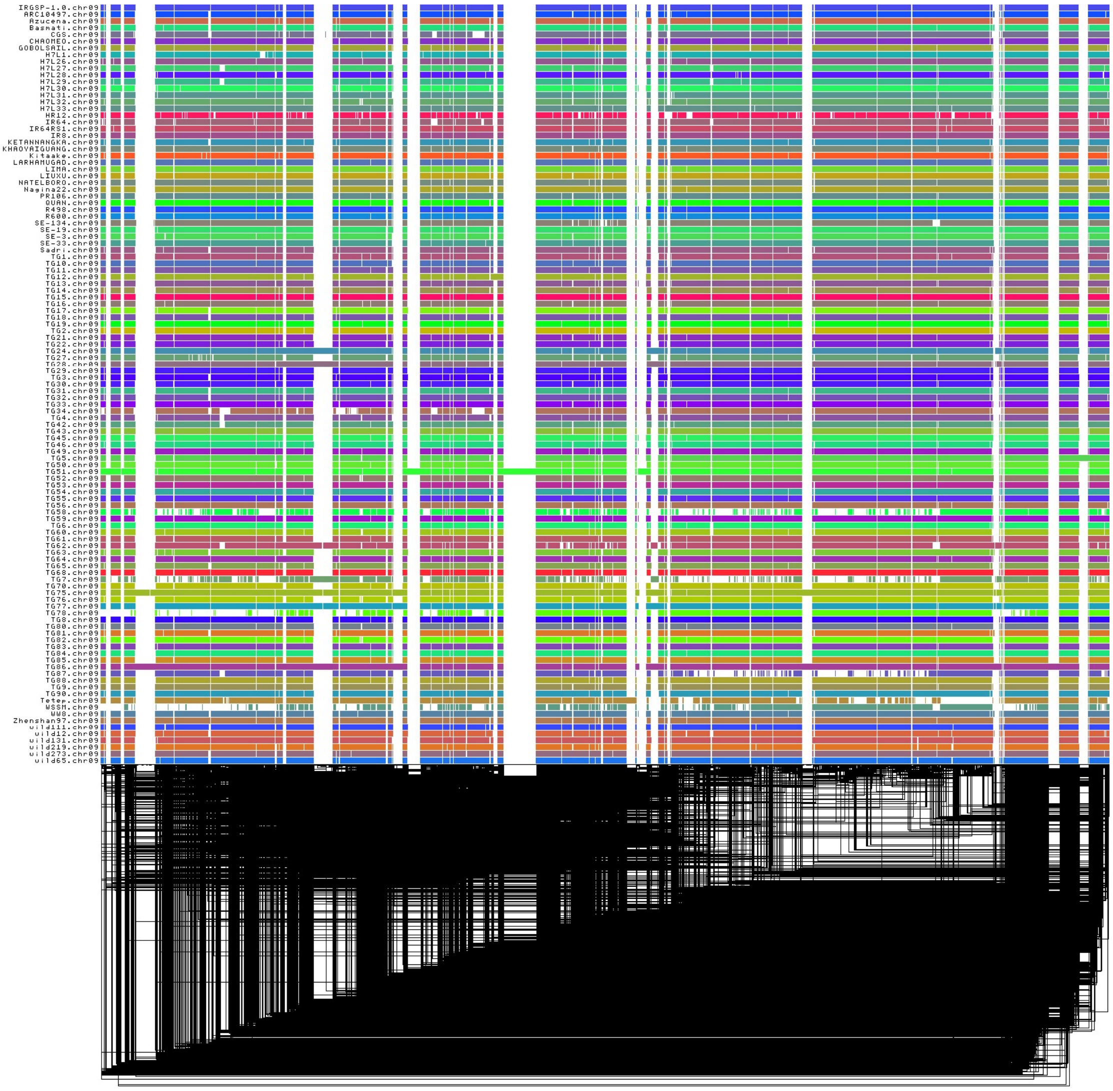

IRGSP-1.0.chr09
ARC10497.chr09
Azucena.chr09
Basmati.chr09
CGS.chr09
CHAOMEO.chr09
GOBOLSAIL.chr09
H7L1.chr09
H7L26.chr09
H7L27.chr09
H7L28.chr09
H7L29.chr09
H7L30.chr09
H7L31.chr09
H7L32.chr09
H7L33.chr09
HR12.chr09
IR64.chr09
IR64RS1.chr09
IR8.chr09
KETANNANGKA.chr09
KHAOYAIGUANG.chr09
Kitaake.chr09
LARHAMUGAD.chr09
LIMA.chr09
LIUXU.chr09
NATELBORO.chr09
Nagina22.chr09
PR106.chr09
QUAN.chr09
R498.chr09
R600.chr09
SE-134.chr09
SE-19.chr09
SE-3.chr09
SE-33.chr09
Sadri.chr09
TG1.chr09
TG10.chr09
TG11.chr09
TG12.chr09
TG13.chr09
TG14.chr09
TG15.chr09
TG16.chr09
TG17.chr09
TG18.chr09
TG19.chr09
TG2.chr09
TG21.chr09
TG22.chr09
TG24.chr09
TG27.chr09
TG28.chr09
TG29.chr09
TG3.chr09
TG30.chr09
TG31.chr09
TG32.chr09
TG33.chr09
TG34.chr09
TG4.chr09
TG42.chr09
TG43.chr09
TG45.chr09
TG46.chr09
TG49.chr09
TG5.chr09
TG50.chr09
TG51.chr09
TG52.chr09
TG53.chr09
TG54.chr09
TG55.chr09
TG56.chr09
TG58.chr09
TG59.chr09
TG6.chr09
TG60.chr09
TG61.chr09
TG62.chr09
TG63.chr09
TG64.chr09
TG65.chr09
TG68.chr09
TG7.chr09
TG70.chr09
TG75.chr09
TG76.chr09
TG77.chr09
TG78.chr09
TG8.chr09
TG80.chr09
TG81.chr09
TG82.chr09
TG83.chr09
TG84.chr09
TG85.chr09
TG86.chr09
TG87.chr09
TG88.chr09
TG9.chr09
TG90.chr09
Tetep.chr09
WSSM.chr09
WW8.chr09
Zhenshan97.chr09
wild111.chr09
wild12.chr09
wild131.chr09
wild219.chr09
wild273.chr09
wild65.chr09

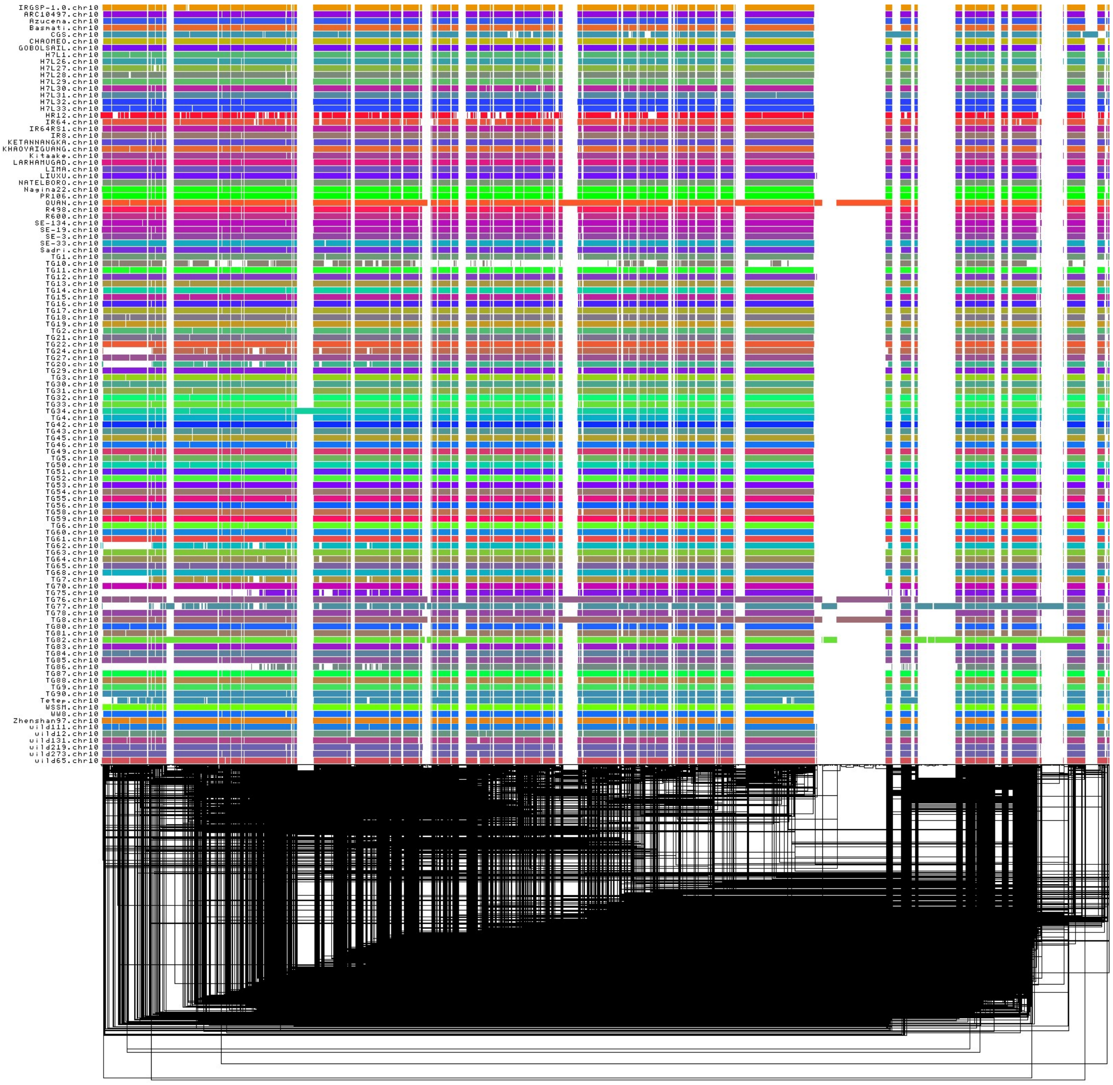
IRGSP-1.0.chr10
ARC10497.chr10
Azucena.chr10
Basmati.chr10
CGS.chr10
CHAOMEO.chr10
GOBOLSAIL.chr10
H7L1.chr10
H7L26.chr10
H7L27.chr10
H7L28.chr10
H7L29.chr10
H7L30.chr10
H7L31.chr10
H7L32.chr10
H7L33.chr10
HR12.chr10
IR64.chr10
IR64RS1.chr10
IR8.chr10
KETANNANGKA.chr10
KHAOYAIGUANG.chr10
Kitaake.chr10
LARHAMUGAD.chr10
LIMA.chr10
LIUXU.chr10
NATELBORO.chr10
Nagina22.chr10
PR106.chr10
QUAN.chr10
R498.chr10
R600.chr10
SE-134.chr10
SE-19.chr10
SE-3.chr10
SE-33.chr10
Sadri.chr10
TG1.chr10
TG10.chr10
TG11.chr10
TG12.chr10
TG13.chr10
TG14.chr10
TG15.chr10
TG16.chr10
TG17.chr10
TG18.chr10
TG19.chr10
TG2.chr10
TG21.chr10
TG22.chr10
TG24.chr10
TG27.chr10
TG28.chr10
TG29.chr10
TG3.chr10
TG30.chr10
TG31.chr10
TG32.chr10
TG33.chr10
TG34.chr10
TG4.chr10
TG42.chr10
TG43.chr10
TG45.chr10
TG46.chr10
TG49.chr10
TG5.chr10
TG50.chr10
TG51.chr10
TG52.chr10
TG53.chr10
TG54.chr10
TG55.chr10
TG56.chr10
TG58.chr10
TG59.chr10
TG6.chr10
TG60.chr10
TG61.chr10
TG62.chr10
TG63.chr10
TG64.chr10
TG65.chr10
TG68.chr10
TG7.chr10
TG70.chr10
TG75.chr10
TG76.chr10
TG77.chr10
TG78.chr10
TG8.chr10
TG80.chr10
TG81.chr10
TG82.chr10
TG83.chr10
TG84.chr10
TG85.chr10
TG86.chr10
TG87.chr10
TG88.chr10
TG9.chr10
TG90.chr10
Tetep.chr10
WSSM.chr10
WW8.chr10
Zhenshan97.chr10
wild111.chr10
wild12.chr10
wild131.chr10
wild219.chr10
wild273.chr10
wild65.chr10

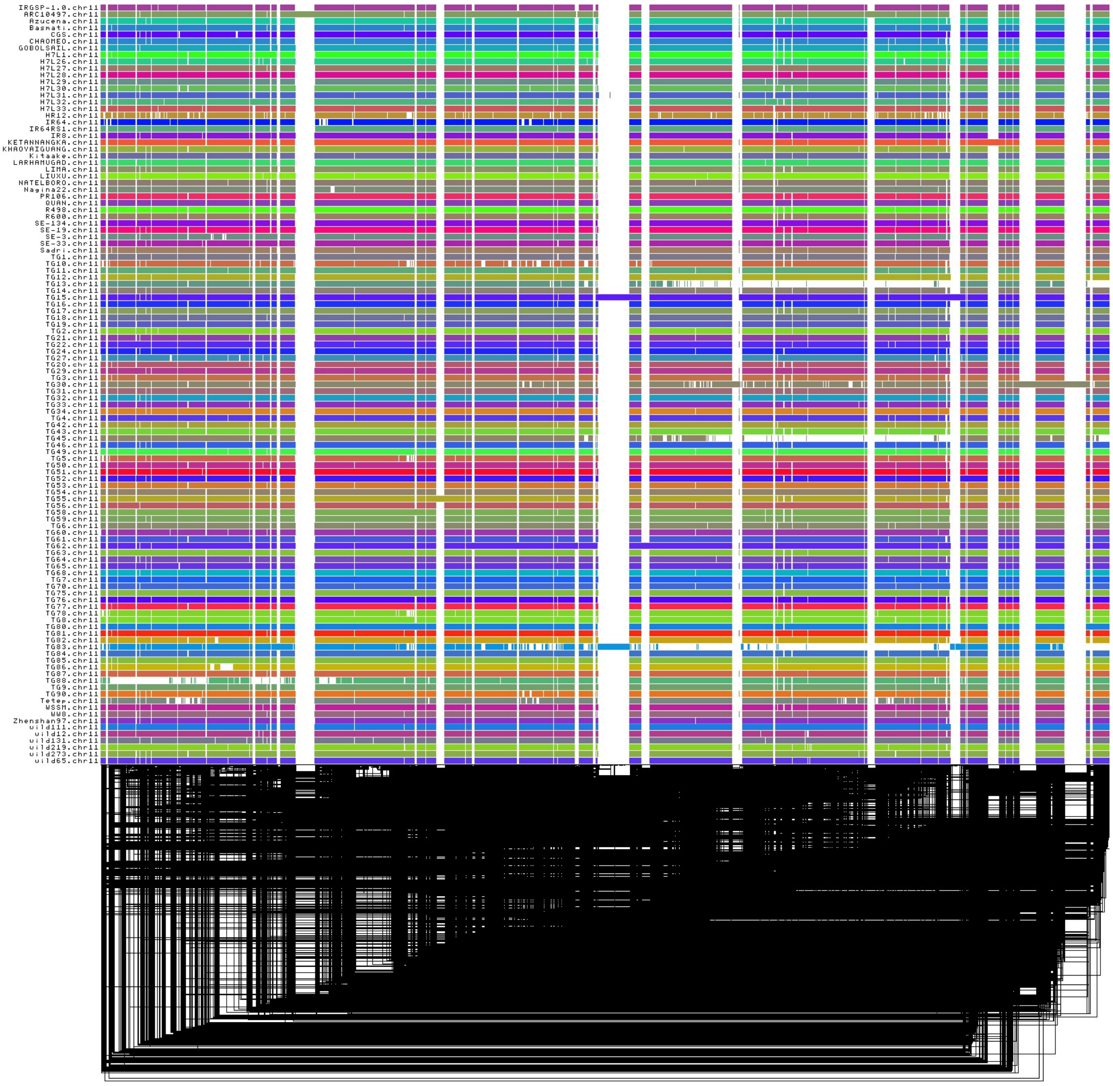

IRGSP-1.0.chr11
ARC10497.chr11
Azucena.chr11
Basmati.chr11
CGS.chr11
CHAOMEO.chr11
GOBOLSAIL.chr11
H7L1.chr11
H7L26.chr11
H7L27.chr11
H7L28.chr11
H7L29.chr11
H7L30.chr11
H7L31.chr11
H7L32.chr11
H7L33.chr11
HR12.chr11
IR64.chr11
IR64RS1.chr11
IR8.chr11
KETANNANGKA.chr11
KHAOYAIGUANG.chr11
Kitaake.chr11
LARHAMUGAD.chr11
LIMA.chr11
LIUXU.chr11
NATELBORO.chr11
Nagina22.chr11
PR106.chr11
QUAN.chr11
R498.chr11
R600.chr11
SE-134.chr11
SE-19.chr11
SE-3.chr11
SE-33.chr11
Sadri.chr11
TG1.chr11
TG10.chr11
TG11.chr11
TG12.chr11
TG13.chr11
TG14.chr11
TG15.chr11
TG16.chr11
TG17.chr11
TG18.chr11
TG19.chr11
TG2.chr11
TG21.chr11
TG22.chr11
TG24.chr11
TG27.chr11
TG28.chr11
TG29.chr11
TG3.chr11
TG30.chr11
TG31.chr11
TG32.chr11
TG33.chr11
TG34.chr11
TG4.chr11
TG42.chr11
TG43.chr11
TG45.chr11
TG46.chr11
TG49.chr11
TG5.chr11
TG50.chr11
TG51.chr11
TG52.chr11
TG53.chr11
TG54.chr11
TG55.chr11
TG56.chr11
TG58.chr11
TG59.chr11
TG6.chr11
TG60.chr11
TG61.chr11
TG62.chr11
TG63.chr11
TG64.chr11
TG65.chr11
TG68.chr11
TG7.chr11
TG70.chr11
TG75.chr11
TG76.chr11
TG77.chr11
TG78.chr11
TG8.chr11
TG80.chr11
TG81.chr11
TG82.chr11
TG83.chr11
TG84.chr11
TG85.chr11
TG86.chr11
TG87.chr11
TG88.chr11
TG9.chr11
TG90.chr11
Tetep.chr11
WSSM.chr11
WW8.chr11
Zhenshan97.chr11
wild111.chr11
wild12.chr11
wild131.chr11
wild219.chr11
wild273.chr11
wild65.chr11

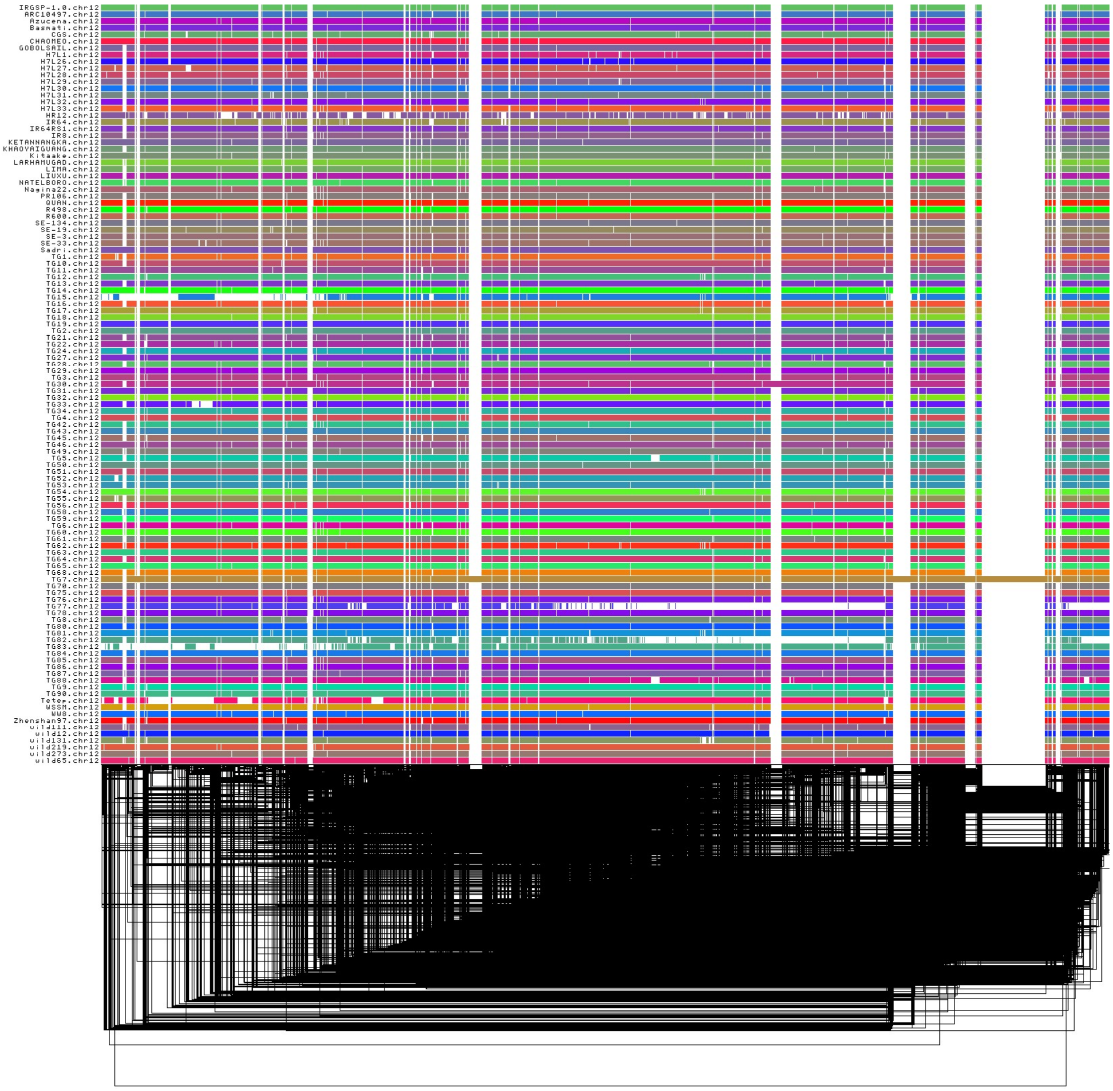
IRGSP-1.0.chr12
ARC10497.chr12
Azucena.chr12
Basmati.chr12
CGS.chr12
CHAOMEO.chr12
GOBOLSAIL.chr12
H7L1.chr12
H7L26.chr12
H7L27.chr12
H7L28.chr12
H7L29.chr12
H7L30.chr12
H7L31.chr12
H7L32.chr12
H7L33.chr12
HR12.chr12
IR64.chr12
IR64RS1.chr12
IR8.chr12
KETANNANGKA.chr12
KHAOYAIGUANG.chr12
Kitaake.chr12
LARHAMUGAD.chr12
LIMA.chr12
LIUXU.chr12
NATELBORO.chr12
Nagina22.chr12
PR106.chr12
QUAN.chr12
R498.chr12
R600.chr12
SE-134.chr12
SE-19.chr12
SE-3.chr12
SE-33.chr12
Sadri.chr12
TG1.chr12
TG10.chr12
TG11.chr12
TG12.chr12
TG13.chr12
TG14.chr12
TG15.chr12
TG16.chr12
TG17.chr12
TG18.chr12
TG19.chr12
TG2.chr12
TG21.chr12
TG22.chr12
TG24.chr12
TG27.chr12
TG28.chr12
TG29.chr12
TG3.chr12
TG30.chr12
TG31.chr12
TG32.chr12
TG33.chr12
TG34.chr12
TG4.chr12
TG42.chr12
TG43.chr12
TG45.chr12
TG46.chr12
TG49.chr12
TG5.chr12
TG50.chr12
TG51.chr12
TG52.chr12
TG53.chr12
TG54.chr12
TG55.chr12
TG56.chr12
TG58.chr12
TG59.chr12
TG6.chr12
TG60.chr12
TG61.chr12
TG62.chr12
TG63.chr12
TG64.chr12
TG65.chr12
TG68.chr12
TG7.chr12
TG70.chr12
TG75.chr12
TG76.chr12
TG77.chr12
TG78.chr12
TG8.chr12
TG80.chr12
TG81.chr12
TG82.chr12
TG83.chr12
TG84.chr12
TG85.chr12
TG86.chr12
TG87.chr12
TG88.chr12
TG9.chr12
TG90.chr12
Tetep.chr12
WSSM.chr12
WW8.chr12
Zhenshan97.chr12
wild111.chr12
wild12.chr12
wild131.chr12
wild219.chr12
wild273.chr12
wild65.chr12
